# Governance in times of war: Public procurement in Ukraine

**DOI:** 10.1371/journal.pone.0305344

**Published:** 2024-06-21

**Authors:** Margaryta Klymak, Tim Vlandas

**Affiliations:** 1 Department of International Development, Kings College London, London, United Kingdom; 2 Department of Social Policy and Intervention, University of Oxford, Oxford, United Kingdom; Thammasat University Institute of East Asian Studies, THAILAND

## Abstract

Wars increase the importance of government functions, yet they also constrain their ability to fulfill these functions. In particular, wars hinder economic activity, thereby limiting governments’ capacity to raise the revenues required to maintain stability and meet the heightened needs of citizens. Effective governance is therefore severely undermined in times of war. However, empirical research on how wars affect government procurement is limited. We address this gap by exploring procurement dynamics using over one million public purchases of goods and services in Ukraine between January 2021 and October 2022, corresponding to the Russian invasion of the country. We document a large fall in the total number of purchases since the invasion and an increase in the share of successfully completed procurements. This higher success rate comes at the cost of efficiency, with the government paying more to source their goods. This can be attributed to the decline in the share of government purchases via online auctions and the reduced competition. Thus, the prioritization of the quick acquisition of goods and services forced governments to sacrifice cost-effectiveness. In summary, the war did not lower the successful purchasing of private goods and services, and transparency was not decreased. However, the trade-off of speed and transparency for greater costs may become increasingly problematic with the growing budget constraints resulting from the war. This article contributes to our understanding of the Ukrainian government choices during the early phase of the war. The results also highlight the importance of ensuring procurement efficiency and transparency when the war ends as reconstruction efforts will require substantial increases in government procurements.

## Introduction

A quarter of the world’s population currently lives in conflict-affected areas [1]. Regardless of their origin, conflicts and wars induce substantial loss of life and damage to key infrastructure, with dire social and economic consequences. Such consequences ultimately undermine economic stability and growth. Conflicts also alter how governments fulfill their roles, particularly in terms of maintaining stability and meeting the heightened expectations of their citizens for protection, public goods, and services. These higher expectations and needs in turn call for even more effective forms of governance. Moreover, inefficient government—firm interactions become not just detrimental to economic efficiency, but may also lead to greater losses on the battlefield and increased suffering for civilians at a time when they are even more reliant on state support.

During wars, governments must purchase goods and services from the private sector, not just to a greater extent than before the war, but also with more limited fiscal capacity and time. This article explores how governments navigate this wartime dilemma by investigating whether and how war reshapes government—firm interactions. We focus on public procurement dynamics, which accounts for 12% of the world’s GDP [2], during one the biggest conflicts in Europe since the end of the Second World War—the ongoing Russian invasion of Ukraine. The conflict started on 24 February 2022 and the resulting economic costs are already substantial. The war is predicted to have led to a 35% real decline in Ukraine’s GDP, which remains 25% below its pre-war level [3]. The estimated damage to the infrastructure is at least 152.5 billion dollars [4]. Going forward, the ongoing military conflict and instability will likely further weaken the Ukrainian economy, likely leading to a pronounced decrease in investment, employment, wages, and productivity. The destruction caused by the war will have long-term consequences, with the Ukrainian government estimating post-war reconstruction costs to exceed $750 billion [5], corresponding to over 3.5 times the pre-war GDP of the country [6]. Once the reconstruction efforts start, a large volume of expenditures will need to be channeled through public procurement into the private sector, either in the form of purchased goods or subcontracted services. The use of public procurement will therefore become even more crucial than it was before the war, when it already amounted to around 15% of the Ukrainian GDP.

The effects of conflicts on economic performance are varied and well-documented [7]. Furthermore, studying public procurement during wars is important as efficient competition in public procurement is difficult to maintain even during times of peace [8]. These challenges are likely exacerbated by wars, possibly creating further issues for governments. On the one hand, governments must quickly obtain goods that may be in limited supply, even in times of peace. On the other hand, the disappearance of many firms can reduce competition for public procurements, while supply-side shocks can increase available prices, and the destruction of key transport and energy infrastructure can prevent the efficient operation of firms.

We investigate procurement dynamics during the Ukrainian war, where the government relies on a uniquely transparent and granular procurement system. After the Euromaidan revolution and the annexation of Crimea in 2014, which marked the beginning of Russia’s invasion of Ukraine, the Ukrainian government collaborated with the private sector and civil society for the development of the procurement platform Prozorro [9]. S5 Appendix in S1 File provides more information on the history and functioning of this procurement system. Prozorro aimed to increase government transparency and efficiency by hosting and monitoring all government procurements in Ukraine in real time. After its launch in 2015, the platform has received numerous awards, such as the Open Government Award [10, 11]. From 2016 onwards, all government authorities were required to use this online procurement system. The Ukrainian government adopted a very transparent public procurement system several years before the war started and recently conducted several prominent anti-corruption raids related to public procurement [12]. However, we lack systematic information on how procurement dynamics have been altered since the war started.

Due to its unparalleled level of detail and transparency, this platform provides a unique opportunity to analyze wartime dynamics related to procurement. We collected information on over one million purchases completed between January 2021 and the end of October 2022. These purchases were carried out by around 40,000 government departments with over 300,000 firms participating. We explore how the invasion displaced the activity of government units and firms in all regions of Ukraine. Our dataset includes a wide range of information across regions and time related to the type of procurement (online or offline), whether it has been successful, and the expected and winning prices paid by the government. This allows us to analyze not just seller and buyer activity and how it adapts to war times, but also price efficiency. The latter is measured by comparing what the government expects to pay (expected value) to the prices it ends up paying (winning value). In addition, we match this information to firm-level data on company size and profits from the Ukrainian IT company YouControl. This allows us to control for buyer characteristics in public procurement and explore firm-level heterogeneity in procurement performance since the war started.

Our analysis yields four main findings. First, the level of procurement activity declined sharply since the start of the war, although there are noteworthy regional differences, and activity levels generally did not recover by summer 2022. Thus, the war has severely and durably disrupted procurement activity. Second, the average purchasing values of purchases decreased, in terms of the expected and winning prices, consistent with the presence of a dual negative shock to prices and quantities during the war. Third, procurements have shifted away from online auctions, which are more cost-efficient but take more time to purchase directly from a supplier, particularly when supply chains are adapting to the disruptions created by the Russian invasion. Fourth, we observe a reduction in the degree of procurement competition determined by the average number of sellers per purchase. The reliance on non-auction procurements together with the lower average competition between sellers jointly led to higher winning ratios (of expected-to-winning prices), accounting for the lower cost efficiency of procurements during the war.

These findings contribute to our understanding of how the procurement sector is adapting to extreme market and security conditions in Ukraine. Faced with tighter budget constraints and urgent demand for goods and services from the private sector, the government appears to have prioritized the speed of procurement at the expense of cost-effectiveness. This finding is crucial for the comprehensive assessment of where the procurement system has performed well and where further improvements may be required. In addition to their policy relevance, our findings shed new light on a novel area of empirical research on the extent to which and how government—firm interactions are reshaped by wars, which represent uniquely large negative economic and security shocks. Our findings contribute to existing literature on the effects of conflicts on economic performance, which are varied and well-documented [7]. For example, studies have focused on GDP [13], state capacity [14], investment [15], growth [16], firm productivity [17], international trade [18–20], and consumption [21]. We provide the first systematic evidence of the effect of wars on public procurement. Furthermore, investigating public procurement during wars is important as even in peaceful times, existing literature has identified a large number of challenges in procurement related to efficient competition [8], the structure of incentives in procurement contracts [22], and transparency and minimizing corruption [23]. Previous research has revealed that conflicts affect firms’ inputs and output prices [24] and disrupt the supply of inputs [25], firm size [26], and survival [27]. Thus, there are good reasons to expect that wars affect firms’ public procurement behavior. Indeed, our results suggest that some of these challenges are likely exacerbated by wars in ways that may create further issues for governments. On the one hand, governments must quickly procure goods that may be in limited supply even in times of peace. On the other hand, the destruction of firms can reduce competition, supply-side shocks can increase available prices, and damage to key infrastructure, transport, and energy can prevent the efficient operation of firms.

## Materials and methods

Our dataset consists of the transactions from Prozorro, the Ukrainian government’s procurement platform, for the period from 01 January 2021 to 31 October 2022. We obtained over 1 million unique purchases before the war and 166,189 unique purchases after the invasion. Prior to 24 February 2022, all government purchases, except for some sensitive categories (e.g. purchases for national security), were required to be processed through—and disclosed on—the procurement platform. Moreover, before the war, online auctions had to be run on Prozorro, while direct purchases from chosen suppliers (reports) had to be reported on Prozorro. Since the full-scale invasion started, the Ukrainian government has briefly removed all previous requirements to report purchases, before re-introducing in June 2022 the mandatory reporting requirements of purchases above the threshold of 50,000 UAH, a relatively low amount equivalent to approximately $1,350 as of the January 2022 exchange rate. In S5 Appendix in S1 File, we provide more information about the history and structure of the procurement system as well as developments during the war. To prevent our results from being driven by these changing dynamics for purchases under the aforementioned threshold, our baseline analyses excluded any purchases below it. However, our results hold if we consider the entire sample consisting of purchases of any amount (S20 Table).

We adopt a fixed effects approach and estimate the following model:

$$y_{imtbfs}=\beta_{0}+\beta_{1}War_{t}+\gamma_{m}+\theta_{t}+\psi_{b}+\;\omega_{f}+\theta_{s}+\epsilon_{imtbfs}$$

where *y*_*imtbfs*_ is an outcome of interest for every purchase *i* occurring in month *m* in year *t* advertised by buyer *b* and approached by firm *f* in industry *s*. Note that while firms can be located nationally and internationally, the procurement platform is used for government purchases only. Therefore, all buyers are based in Ukraine. Coefficient *β*_1_ captures the effect of the Russian invasion. The dummy variable *War* takes a value of 1 if the purchase took place after 24 February 2022, and 0 otherwise. This coefficient therefore captures the effect of the procurement taking place during wartime compared to pre-invasion purchases.

Next, we control for firm fixed effects to capture time-invariant firm characteristics such as their bidding strategies, differences in costs, and owner background. We also conduct additional analyses with high-quality information about firm characteristics collected by YouControl. Government departments (i.e., buyers) fixed effects are included to account for time-invariant differences in buyers (e.g., due to staff, procedures, and size). As robustness checks, we also rerun our analyses with region-fixed effects. In addition to differences between buyers and sellers, cyclical and temporal variation may be present in the content, volume, and characteristics of procurement. Based on this, we also include month *m* and year *to* fixed effects. Next, as procurement behavior could also vary by industry, we include the industry *s* fixed effects. Finally, although the standard errors are clustered at the buyer level in our baseline analyses, the results are robust to using seller and purchase clustering.

Five outcomes of interest are considered in our analysis: 1) the expected prices that the government anticipates paying for their purchases, which they must disclose before the procurement tender is announced; 2) the winning prices that the government ends up paying at the end of the procurement process; 3) the ratio winning value-to-expected value ratio [28], where a higher ratio indicates that winning and expected prices are dissimilar, and hence the government pays more than anticipated, while a lower ratio denotes similar winning and expected prices, and the government thus obtains a good price relative to their expectations, achieving budget savings; 4) a dummy variable that equals 1 if the purchase was successfully completed, and 0 if it failed; and 5) the number of competitors per procedure in our sample, which ranges from 1 to about 30. For ease of comparison, we standardize all variables but also provide the results with non-standardized variables in S2 Appendix in S1 File.

## Results

**Fig 1:**
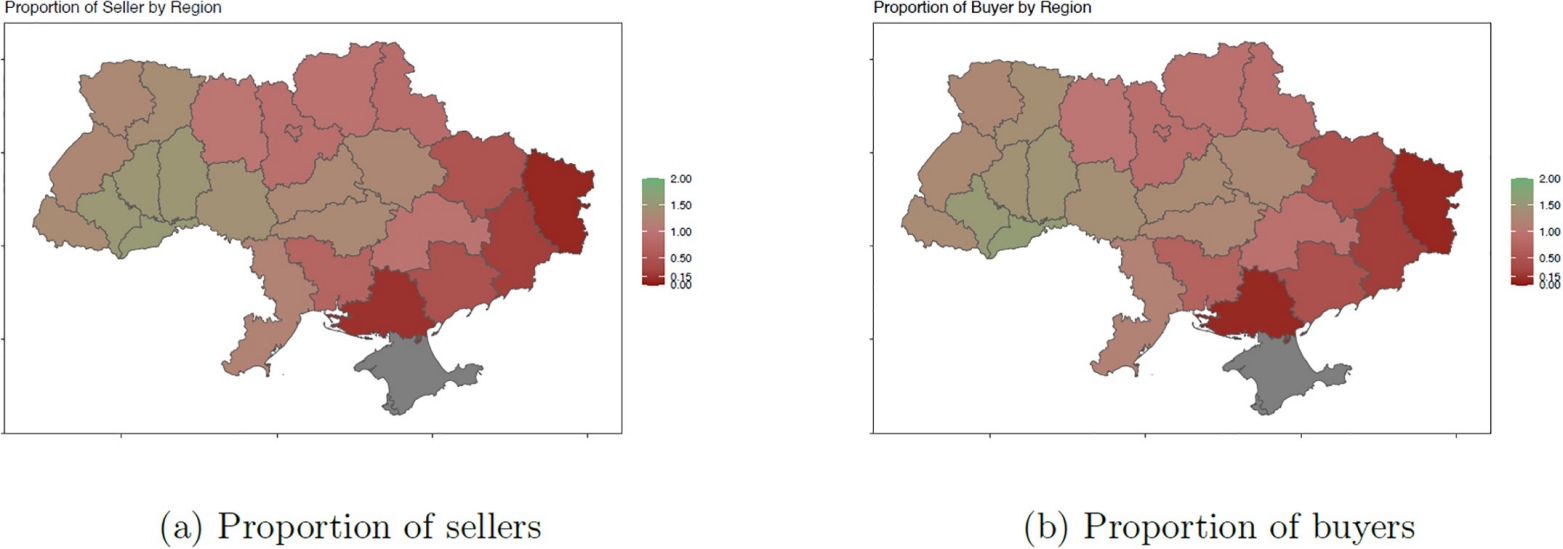
Proportion of sellers and buyers before and during the war. The left panel plots the proportion of sellers in each region during the war divided by the proportion of sellers in each region before the war. For example, a ratio of 2 indicates that the proportion of sellers in that region was twice as high during the war compared to before the war. The right panel presents the same information for buyers. The period before the war starts from the January 2021 until February 2022, while the period during the war runs from 24 February 2022 until the end of October 2022. Note that S4 and S5 Figs show the data or the time before and during the war as well as with and without population weighing, respectively.

Fig 1 presents the impact of the war on procurement dynamics across regions in Ukraine. The proportions of unique buyers (i.e., government departments) and unique sellers (i.e., firms) before and after the war are compared by dividing the regional proportions during the war by proportions prior to the invasion. Regions in dark red denote ratios under 1, indicating that activity after the war started was much lower than before, while regions in green experienced a higher proportion of buying and selling after the war. The procurement activity intensity appears to have been restructured across various parts of the country. Before the war, the majority of buying and selling activity was concentrated in Kyiv, with a slightly higher concentration in the western and central regions, and much lower levels in the east (S5 Fig). In contrast, after the invasion, the proportion of seller activity and buyer demand shifted away from the east and toward the west, southwest, some central regions, and to a lesser extent, some center east regions.

**Fig 2:**
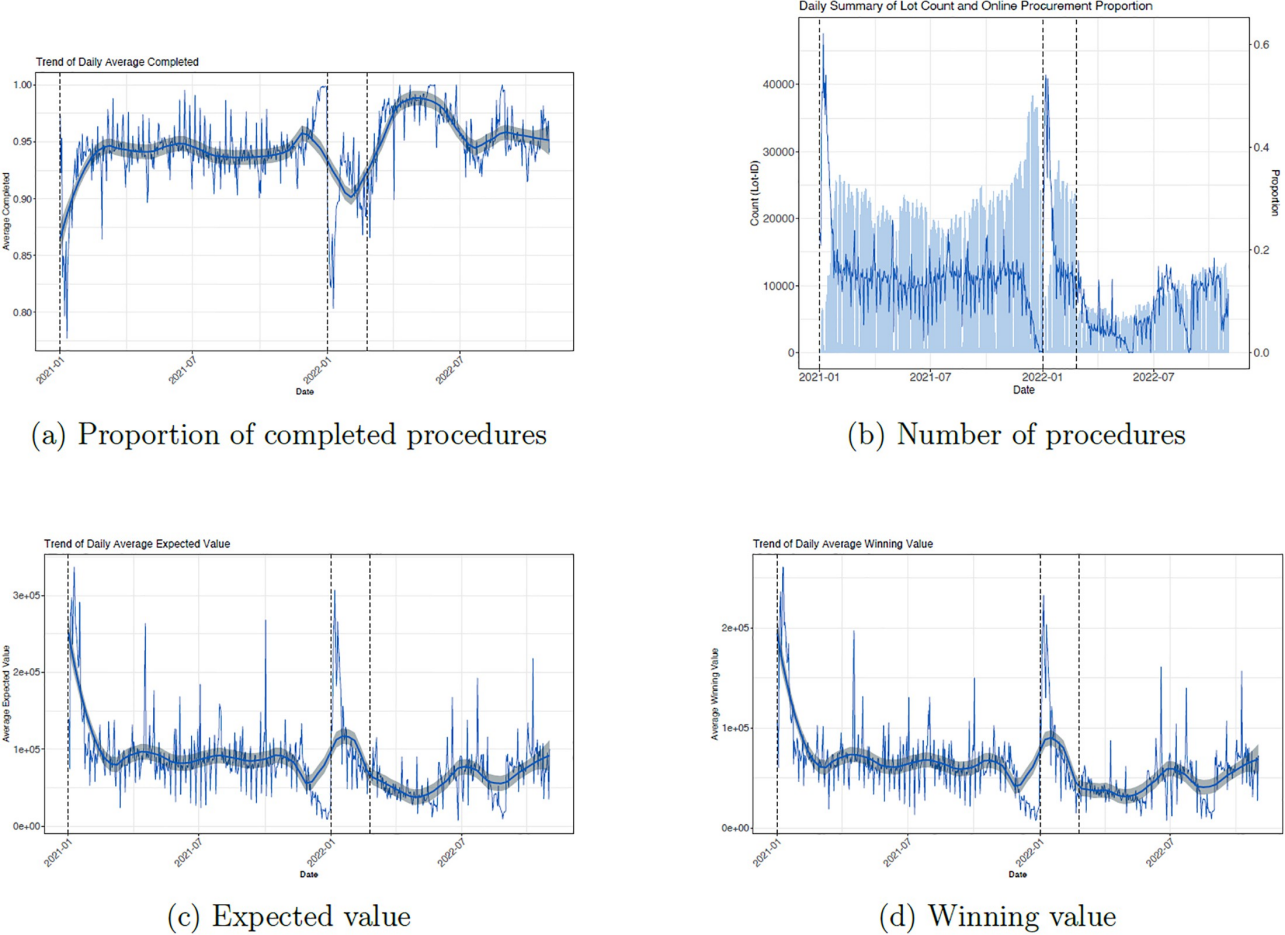
Procurement dynamics over time. (a) Number of procedures, (b) their completion rate, (c) expected value, and (d) winning value before (01 January 2021–23 February 2022) and during the war (24 February 2022–31 October 2022). The black vertical line indicates the beginning of the year, while the dashed line indicates the beginning of the invasion. The proportion of completed procedures denotes the percentage of lots that were successfully completed as opposed to failed or canceled lots. The number of procedures is the number of lots advertised per day. The expected and winning values are the average amounts the Ukrainian government is willing to pay and the resulting winning amounts, respectively.

As the war unfolded, the government faced multiple constraints. In particular, the mix of goods and services that it had to buy was different from that of pre-war times, while supply chains were disrupted. To determine how the government reacted to these challenges, we explore how the type and volume of procurements were affected by the war, and how the prices that governments expected to pay, and then ended up paying, were adversely affected by the invasion. Fig 2 plots the proportion of successfully completed procedures, daily purchase activity, the prices anticipated by the government, and the winning prices, respectively, for the study period. The proportion of successfully completed procedures is defined as the proportion of government procurement purchases that were advertised and then successfully completed (as opposed to either failing or canceled purchases). The daily purchase activity is the total number of new purchases released by the government on a given day. The anticipated price denotes the cost determined by the government given the costs of similar goods on the market. This anticipated price needs to be indicated when the advertisement for a tender is released and serves as a guide for sellers. The winning price is the final price offered by the sellers who were awarded the contract. Following the Russian invasion, the success rate of procedures increased sharply from 93% before the war to 97% just after the war, and subsequently stabilized around 95% over the summer (Fig 2(a)). Thus, the government appeared to become more successful at managing the completion of purchases. However, this occurred in a wider context where the daily activity declined by more than 50% straight after the invasion began and did not return to its initial pre-war levels, as shown on the vertical axis on the left side of Fig 2(b). This suggests that government authorities prioritized their most important and urgent goods and services.

All government agencies must choose between two types of procurement: online auctions, which tend to be more efficient as they aim to achieve higher value for money by attracting more competition; or the ‘offline’ procurement method, in which a government can select a supplier directly, thereby minimizing the time it takes to complete the process. In arbitrating between the cost and the time constraints of these two types of procurement, government buyers appear to have prioritized the latter, as shown by the falling proportion of online auctions as the war unfolded, as shown on the vertical axis on the right side of Fig 2(b). In addition, governments need to consider the prices that they expect to pay and those that they end up paying. Fig 2(c) and 2(d) compare anticipated and actual prices in the year before the war (February—October 2021) and the year since the war started. Both panels suggest that the values slightly declined, partially reflecting the necessary budget limitations due to the war. S3 Fig provides information for other procurement dynamics, while S6 Fig reproduces Fig 2 by changing the threshold for which purchases are included in the analysis.

**Fig 3:**
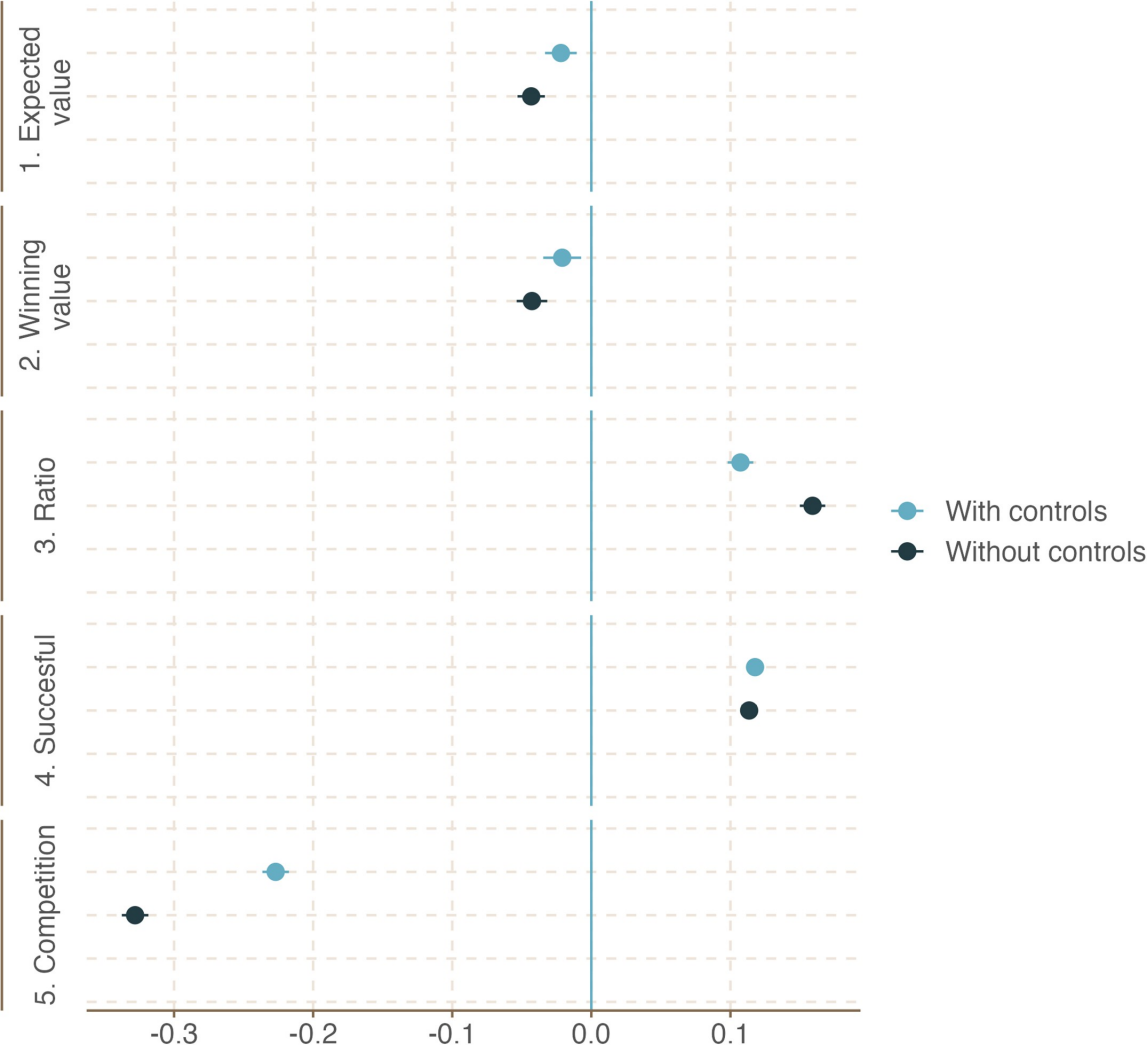
Regression results. The coefficients were obtained from separate regressions performed with and without controls (S1 and S2 Tables, respectively). All dependent variables were standardized to allow for direct comparisons between the effect of the war across all outcomes. The expected and winning values are the average amounts per lot the Ukrainian government is willing to pay and the resulting winning amounts, respectively. The ratio refers to the ratio of winning and expected values. The proportion of completed procedures is the percentage of lots that were successfully completed as opposed to failed or canceled lots. Competition is the average number of competitors per lot.

While the war appears to have affected the temporal and geographical distribution of procurement volumes, types, and prices, these effects could be capturing various unobserved confounders. To systematically explore these dynamics, we present results from several regressions on procurement value, success, and competition during the war. We include a wide range of fixed effects, not just at the month and year level, but also at the buyer and seller levels. We also perform further robustness checks with region-fixed effects and additional controls for firms and regions, reported in the Supporting Information. Fig 3 displays our fixed effects regression estimates for the following standardized outcomes of interest: expected and winning values; the winning value-to-expected value ratio; the successful completion purchases; and the number of competitors per purchase. S1 Table reports the full results. The effect of the war on all the outcomes is statistically significant. More specifically, the invasion was associated with lower expected values and competition, yet it increased the price ratio and success rate. Thus, government buyers achieved better outcomes in terms of completing their procurements. However, this came at the cost of lower competition and cost-effectiveness.

We performed several robustness checks, with the results presented in the Supporting Information. First, S1 Table reveals how the results change when we include a different set of fixed effects, with the last column corresponding to the coefficients presented in Fig 2. The table indicates a decline in the reliance on auctions following the war, thereby corroborating our earlier descriptive result that government authorities prioritized the speed provided by non-auction procurements over the better prices generated by online auctions. We have employed standardized dependent variables throughout the analysis. S21 Table replicates all specifications from S1 Table using non-standardized dependent variables. Adopting non-standardized dependent variables allows us to apply a logarithmic transformation of the expected and winning values. Second, as reported in S2 Table, the results are stable when controlling for a major policy change that occurred after the war had started (see S5 Appendix in S1 File). Similarly, changing the threshold that determines which transactions are included in our analyses does not change our results (S20 Table). Third, we explore whether the effect of the war was different between the initial and later stages. S4 Table suggests that most of the effects became more pronounced as the war unfolded. For example, the effect on expected and winning values only became statistically significant in the later period, while the magnitude of the effect generally increased over time for most other outcomes.

Next, we examine whether our results are driven by region-specific dynamics. We find that excluding the capital regions does not change the results (S5 Table), nor does excluding the regions Luhansk and Donetsk, which were closest to the location of the war (S6 and S7 Tables). This remains the case even when excluding other particularly exposed neighboring regions (S8 and S9 Tables). Similarly, controlling for region-fixed effects or population does not change the results (S17 to S19 Tables). We then restrict our sample to homogenous goods only (S10 Table) following the approach described in Klymak and Baumann [28], revealing that the findings are not driven by changes in the composition of goods bought by the government. S22 Table reports the results without the standardized dependent variables. We then evaluate whether the effects of the war on competition change if we restrict our dataset only to online auctions. The results show that the war increased competition within online auctions. This suggests that the overall negative effect indicates a shift away from online auctioning (where competition increased) and toward no competition (S11 Table).

Moreover, we investigate several other dimensions of heterogeneity. First, we assess whether there is heterogeneity for different procurement types (goods, services, and work). We find that our results hold for goods, while differences are observed for services and work. More specifically, for services, the war did not exert a statistically significant effect on values, while for work, the war resulted in overall higher competition (S12 Table). We then show that our overall results are robust when including controls for types of contracted objects (S13 Table). Following this, we match uniquely granular and high-quality firm-level data from YouControl to procurement seller tax identifiers to analyze heterogeneity across firms of different profit levels and sizes. We find that the negative effect of the war on expected and winning values were stronger for more profitable firms, and its positive effect on the ratio was more pronounced for profitable firms, while there were no differences in competition and success rates (S14 Table). We also find evidence for differences across firm size, whereby the effects of war appeared in many instances more pronounced for medium firms compared to small and micro firms (S15 and S16 Tables).

Finally, we conduct two jackknife tests for all dependent variables, revealing that results for all variables, except for the success rate, are robust. For this test, we replicate the main specification with all fixed effects while dropping each region of Ukraine in turn. S1 Fig plots the coefficients from the regressions. The findings demonstrate that the vast majority of coefficients are centered around the coefficient found in our main results. For our second jackknife test, we dropped each week in our dataset in turn (S2 Fig). The procurement dataset allows us to choose two reporting dates: the date when the intended purchase is advertised; or the date when the purchase was completed. Our baseline results are based on the latter measure. As a robustness check, we confirm in S3 Table that the effect of the war on our variables holds if we adopt the date on which the purchase was advertised.

## Discussion

In the past two decades, governments have come under increasing pressure to rapidly adapt their operations to various crises, from the 2008 financial crisis to the Covid-19 pandemic, and most recently, Russia’s invasion of Ukraine. Effective governance in public functions has a crucial role to play, in terms of the management of these crises and selecting the best policy responses to these emergencies [4]. The procurement system of a government plays a key role in how they pre-empt and react to crises, allowing them to efficiently purchase goods and contract out services to achieve public goals.

This article provides the first systematic empirical analysis of procurement behavior during wars, which represent particularly large dual shocks to the economic and national security of many countries around the world. Our analyses rely on a uniquely granular dataset that allows us to not only measure whether the government purchase took place, but also a wide range of other outcomes that are particularly relevant during times of conflict, such as procurement type, seller competition, and price efficiency. We can observe the precise time and place of each procurement and thus determine whether the purchase took place after the invasion and control for unobservable geographical and temporal heterogeneity.

Our analysis increases our understanding of how governments react to large shocks that disrupt supply chains and national security. More specifically, our results suggest that the average price per purchase, online activity, and competition declined after the Russian invasion of Ukraine. This implies that government authorities prioritized speed at the cost of greater prices. Consistent with this latter interpretation, we find that competition in online purchases even increased after the war began, and thus overall competition declined because of the growing reliance on offline procurements (S6 Table). These findings uncover for the first time how the price and speed of procurement provision react to the shocks associated with wars. This observation can aid policymakers to channel large financial resources efficiently and quickly through the Ukrainian procurement system for the reconstruction of the economy after the war.

## Supporting information

S1 Table Effect of the war—Full results plotted in Fig 3. Standard errors clustered at the buyer level are shown in brackets; *p < 0.1; **p < 0.05; ***p < 0.01. Clustering at the seller level does not change our conclusions. Each panel presents the results from ordinary least squares (OLS) regressions for a different dependent variable, while each column presents the results when changing the fixed effect structures adopted. All dependent variables have been standardized.
(PNG)

S2 Table Effect of the war when controlling for policy changes (1). Standard errors clustered at the buyer level are shown in brackets; *p < 0.1; **p < 0.05; ***p < 0.01. Each panel presents the results from OLS regressions for a different dependent variable, while each column presents the results when changing the fixed effect structures adopted. All dependent variables have been standardized. S5 Appendix in S1 File provides more information about the policy change.
(PNG)

S3 Table Effect of the war when controlling for policy changes (2). Standard errors clustered at the buyer level are shown in brackets; *p < 0.1; **p < 0.05; ***p < 0.01. Each panel presents the results from OLS regressions for a different dependent variable, while each column presents the results when changing the fixed effect structures adopted. All dependent variables have been standardized. S5 Appendix in S1 File presents more information about the policy change.
(PNG)

S4 Table Effect of the war during the early and late war stages. Standard errors clustered at the buyer level are shown in brackets; *p < 0.1; **p < 0.05; ***p < 0.01. Each column presents the results from OLS regressions for a different dependent variable. The top panel restricts the sample to the early stages of the war (first two months), while the bottom panel does the same but for the later stages of the war. All columns include all fixed effects. All dependent variables have been standardized.
(PNG)

S5 Table Effect of the war when excluding observations for the capital region. Standard errors clustered at the buyer level are shown in brackets; *p < 0.1; **p < 0.05; ***p < 0.01. Each panel presents the results from OLS regressions for a different dependent variable, while each column presents the results when changing the fixed effect structures adopted. All dependent variables have been standardized.
(PNG)

S6 Table Effect of the war when excluding observations for the Luhansk and Donetsk regions (1). Standard errors clustered at the buyer level are shown in brackets; *p < 0.1; **p < 0.05; ***p < 0.01. Each panel presents the results from OLS regressions for a different dependent variable, while each column presents the results when changing the fixed effect structures adopted. All dependent variables have been standardized.
(PNG)

S7 Table Effect of the war when excluding observations for the Luhansk and Donetsk regions (2). Standard errors clustered at the buyer level are shown in brackets; *p < 0.1; **p < 0.05; ***p < 0.01. Each panel presents results from OLS regressions for a different dependent variable, while each column presents the results when changing the fixed effect structures adopted. All dependent variables have been standardized.
(PNG)

S8 Table Effect of the war when excluding observations for Luhansk, Donetsk, and the neighboring regions (1). Standard errors clustered at the buyer level are shown in brackets; *p < 0.1; **p < 0.05; ***p < 0.01. Each panel presents the results from OLS regressions for a different dependent variable, while each column depicts the results when changing the fixed effect structures adopted. All dependent variables have been standardized.
(PNG)

S9 Table Effect of the war when excluding observations for Luhansk, Donetsk, and the neighboring regions (2). Standard errors clustered at the buyer level are shown in brackets; *p < 0.1; **p < 0.05; ***p < 0.01. Each panel presents the results from OLS regressions for a different dependent variable, while each column presents the results when changing the fixed effect structures adopted. All dependent variables have been standardized.
(PNG)

S10 Table Effect of the war restricting observations to homogenous goods only. Standard errors clustered at the buyer level are shown in brackets; *p < 0.1; **p < 0.05; ***p < 0.01. Each panel presents the results from OLS regressions for a different dependent variable, while each column presents the results when changing the fixed effect structures adopted. All dependent variables have been standardized.
(PNG)

S11 Table Effect of the war for sample restricted to online auctions only. Standard errors clustered at the buyer level are shown in brackets; *p < 0.1; **p < 0.05; ***p < 0.01. Each column presents the results from OLS regressions for a different dependent variable while restricting the sample to online auctions only. Full fixed effects are included. All dependent variables have been standardized.
(PNG)

S12 Table Effect of the war for separate samples by types of goods purchased. Standard errors clustered at the buyer level are shown in brackets; *p < 0.1; **p < 0.05; ***p < 0.01. Each column presents the results from OLS regressions for a different dependent variable, while each panel captures a different sample restriction for goods, services, and work, respectively. All regressions included full fixed effects. All dependent variables have been standardized.
(PNG)

S13 Table Effect of the war while controlling for different types of goods purchased. Standard errors clustered at the buyer level are shown in brackets; *p < 0.1; **p < 0.05; ***p < 0.01. Each column presents the results from OLS regressions for a different dependent variable. The top panel reproduces our baseline result while the bottom panel controls for type of purchase. Full fixed effects are included. All dependent variables have been standardized.
(PNG)

S14 Table Effect of the war for firms with different pre-war profits. Standard errors clustered at the buyer level are shown in brackets; *p < 0.1; **p < 0.05; ***p < 0.01. Each column presents the results from OLS regressions for a different dependent variable. We further include an interaction term between our dummy variable war and a measure of firm profits collected by YouControl. Full fixed effects are included. All dependent variables have been standardized.
(PNG)

S15 Table Effect of the war by firm size (1). Standard errors clustered at the buyer level are shown in brackets; *p < 0.1; **p < 0.05; ***p < 0.01. Each panel presents the results from OLS regressions for a different dependent variable, while each column presents results when changing the fixed effect structures adopted. All dependent variables have been standardized.
(PNG)

S16 Table Effect of the war by firm size (2). Standard errors clustered at the buyer level are shown in brackets; *p < 0.1; **p < 0.05; ***p < 0.01. Each panel presents the results from OLS regressions for a different dependent variable, while each column presents the results when changing the fixed effect structures adopted. All dependent variables have been standardized.
(PNG)

S17 Table Effect of the war controlling for regions (1). Standard errors clustered at the buyer level are shown in brackets; *p < 0.1; **p < 0.05; ***p < 0.01. Each panel presents the results from OLS regressions for a different dependent variable, while each column presents results when changing the fixed effect structures adopted. All dependent variables have been standardized.
(PNG)

S18 Table Effect of the war controlling for regions (2). Standard errors clustered at the buyer level are shown in brackets; *p < 0.1; **p < 0.05; ***p < 0.01. Each panel presents the results from OLS regressions for a different dependent variable, while each column presents the results when changing the fixed effect structures adopted. All dependent variables have been standardized.
(PNG)

S19 Table Effect of the war controlling for region population. Standard errors clustered at the buyer level are shown in brackets; *p < 0.1; **p < 0.05; ***p < 0.01. Each panel presents the results from OLS regressions for a different dependent variable, while each column presents the results when changing the fixed effect structures adopted. All dependent variables have been standardized. This table controls for region population to account for the potential non-linear scaling of procurement activity with population size.
(PNG)

S20 Table Effect of the war based on the tender date for the whole sample. Standard errors clustered at the buyer level are shown in brackets; *p < 0.1; **p < 0.05; ***p < 0.01. Each panel presents the results from OLS regressions for a different dependent variable, while each column presents the results when changing the fixed effect structures adopted. All dependent variables have been standardized. The sample includes purchases of any value.
(PNG)

S21 Table Effect of the war. Standard errors clustered at the buyer level are shown in brackets; *p < 0.1; **p < 0.05; ***p < 0.01. Each panel presents the results from OLS regressions for a different dependent variable, while each column presents the results when changing the fixed effect structures adopted.
(PNG)

S22 Table Effect of the war, restricting samples to homogenous goods. Standard errors clustered at the buyer level are shown in brackets; *p < 0.1; **p < 0.05; ***p < 0.01. Each panel presents the results from OLS regressions for a different dependent variable, while each column presents the results when changing the fixed effect structures adopted.
(PNG)

S23 Table Effect of the war on values based on publishing date. Standard errors clustered at the buyer level are shown in brackets; *p < 0.1; **p < 0.05; ***p < 0.01. Each panel presents results from the OLS regressions for a different dependent variable, while each column depicts the results when changing the fixed effect structures adopted. Every procurement transaction has two reference dates: the tender date captured when the purchase occurs; and the publishing date referring to the time the buyer posted the call for tender. Our baseline regressions throughout the analyses use the tender dates as the reference time, and we reproduce our analyses with the publication date in this regression.
(PNG)

S1 Fig Jackknife results for regions. Jackknife test results for regions in Ukraine. We drop each of the 24 Ukraine regions in turn to determine the coefficients. The pre-was period is from January 2021 until February 2022, while the post-war period is from 24 February 2022 until the end of October 2022.
(PNG)

S2 Fig Jackknife results for weeks. Jackknife test results for weeks. We drop each week in each year in our dataset in turn to determine the coefficients. The pre-was period is from January 2021 until February 2022, while the post-war period is from 24 February 2022 until the end of October 2022.
(PNG)

S3 Fig Other procurement dynamics. (PNG)

S4 Fig Proportion of sellers and buyers before and during the war (weighed by regional population). Proportion of sellers and buyers in each region of Ukraine before and during the war, weighed by the population proportion in each of these regions, respectively. The pre-was period is from January 2021 until February 2022, while the post-war period is from 24 February 2022 until the end of October 2022.
(PNG)

S5 Fig Proportion of sellers and buyers before and during the war. Proportion of sellers and buyers in each region of Ukraine before and during the war. The pre-was period is from January 2021 until February 2022, while the post-war period is from 24 February 2022 until the end of October 2022.
(PNG)

S6 Fig Procurement dynamics over time for purchases above 50,000 UAH. Proportion of sellers and buyers in each region of Ukraine before and during the war weighted by population. The pre-was period is from January 2021 until February 2022, while the post-war period is from 24 February 2022 until the end of October 2022. The black and vertical lines indicate the beginning of the year and the invasion, respectively.
(PNG)

S1 File S1 Appendix. Results with standardized dependent variables. S1.1 Jackknife results. S2 Appendix. Results with non-standardized dependent variables. S2.1 Tender date. S2.2 Publishing date. S3 Appendix. Other results. S4 Appendix. Results for procurement above 50,000. S5 Appendix. Procurement system.
(DOCX)
