# Supplementary figures and images for: Governance in times of war: Public procurement in Ukraine
Source: PLoS One. 2024 Jun 21;19(6):e0305344. doi: 10.1371/journal.pone.0305344 (PMC11192321; doi:10.1371/journal.pone.0305344)

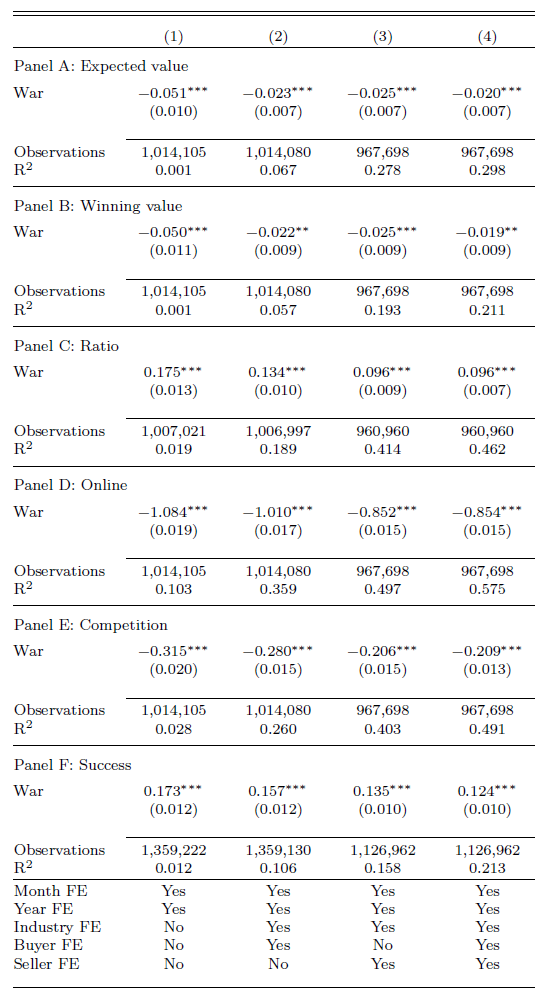

Supplement: S1 Table — Standard errors clustered at the buyer level are shown in brackets; *p < 0.1; **p < 0.05; ***p < 0.01. Clustering at the seller level does not change our conclusions. Each panel presents the results from ordinary least squares (OLS) regressions for a different dependent variable, while each column presents the results when changing the fixed effect structures adopted. All dependent variables have been standardized. (PNG) [file pone.0305344.s001.png]

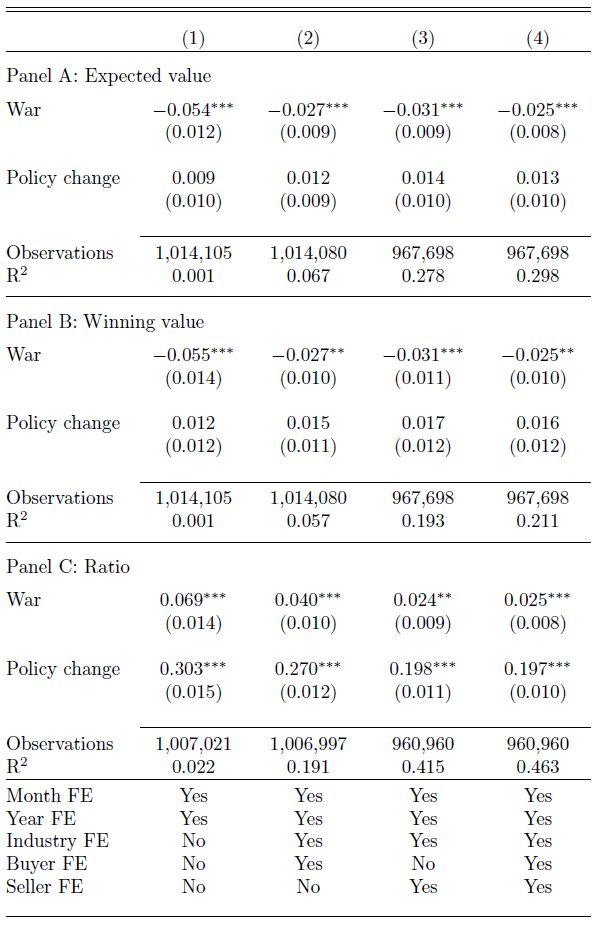

Supplement: S2 Table — Standard errors clustered at the buyer level are shown in brackets; *p < 0.1; **p < 0.05; ***p < 0.01. Each panel presents the results from OLS regressions for a different dependent variable, while each column presents the results when changing the fixed effect structures adopted. All dependent variables have been standardized. S5 Appendix in S1 File provides more information about the policy change. (PNG) [file pone.0305344.s002.png]

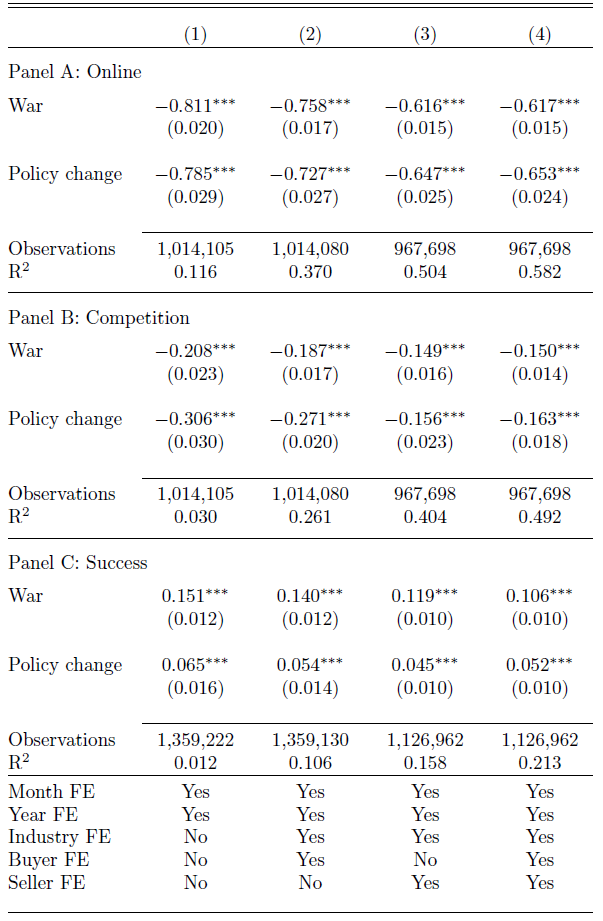

Supplement: S3 Table — Standard errors clustered at the buyer level are shown in brackets; *p < 0.1; **p < 0.05; ***p < 0.01. Each panel presents the results from OLS regressions for a different dependent variable, while each column presents the results when changing the fixed effect structures adopted. All dependent variables have been standardized. S5 Appendix in S1 File presents more information about the policy change. (PNG) [file pone.0305344.s003.png]

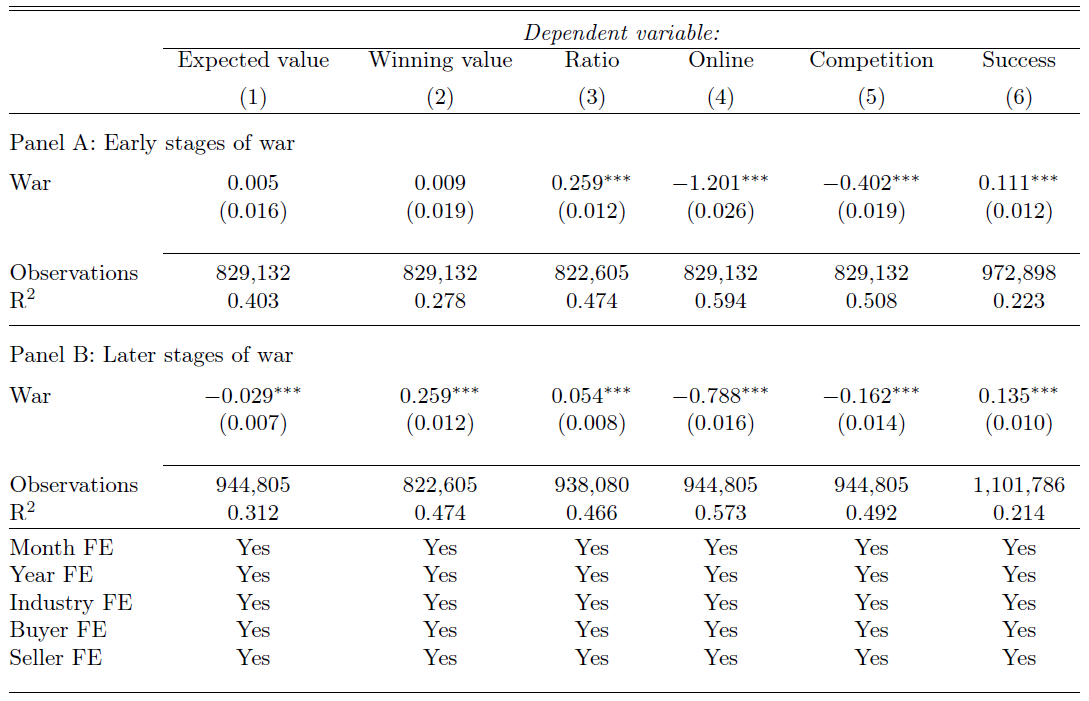

Supplement: S4 Table — Standard errors clustered at the buyer level are shown in brackets; *p < 0.1; **p < 0.05; ***p < 0.01. Each column presents the results from OLS regressions for a different dependent variable. The top panel restricts the sample to the early stages of the war (first two months), while the bottom panel does the same but for the later stages of the war. All columns include all fixed effects. All dependent variables have been standardized. (PNG) [file pone.0305344.s004.png]

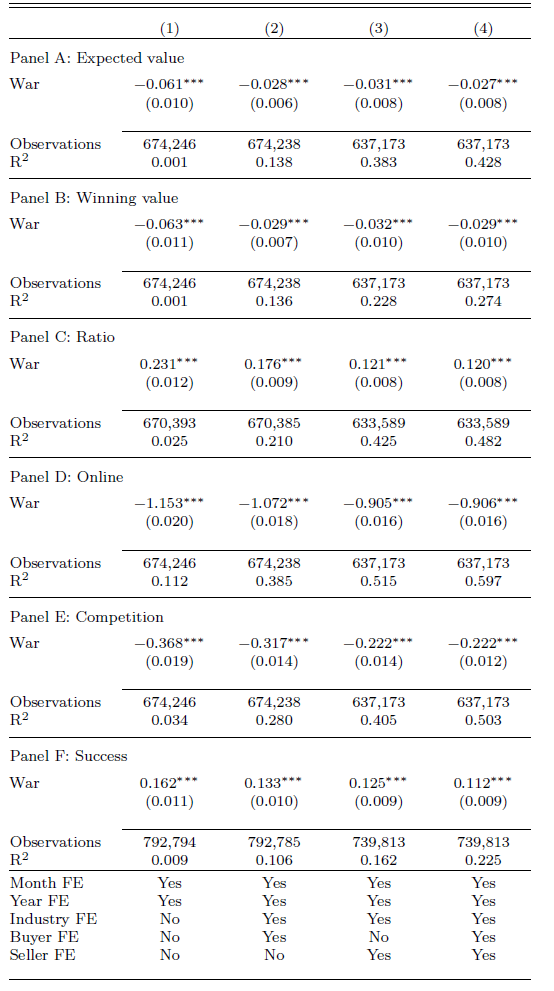

Supplement: S5 Table — Standard errors clustered at the buyer level are shown in brackets; *p < 0.1; **p < 0.05; ***p < 0.01. Each panel presents the results from OLS regressions for a different dependent variable, while each column presents the results when changing the fixed effect structures adopted. All dependent variables have been standardized. (PNG) [file pone.0305344.s005.png]

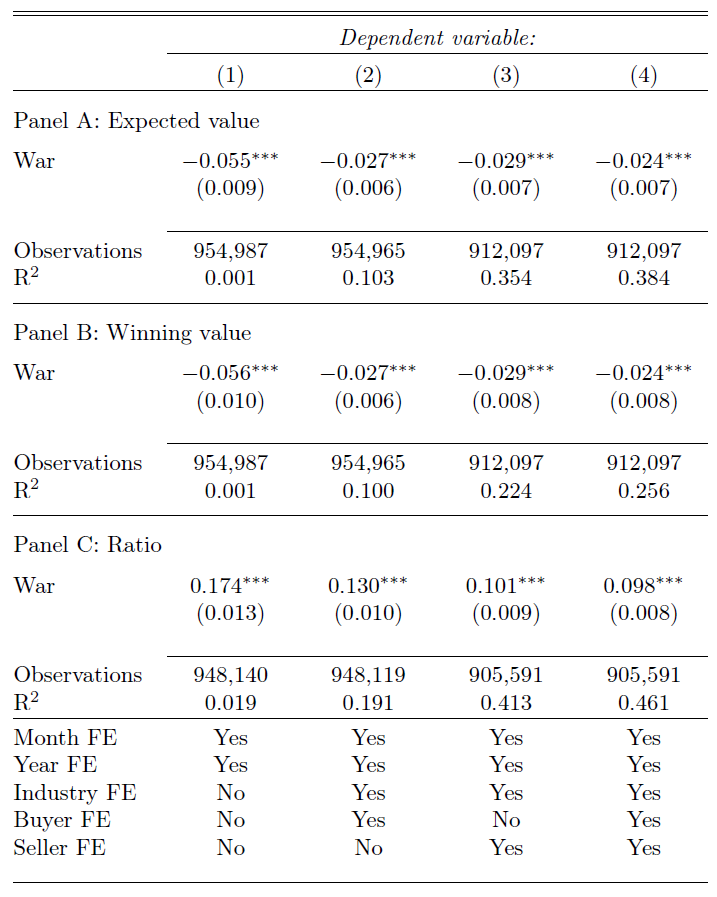

Supplement: S6 Table — Standard errors clustered at the buyer level are shown in brackets; *p < 0.1; **p < 0.05; ***p < 0.01. Each panel presents the results from OLS regressions for a different dependent variable, while each column presents the results when changing the fixed effect structures adopted. All dependent variables have been standardized. (PNG) [file pone.0305344.s006.png]

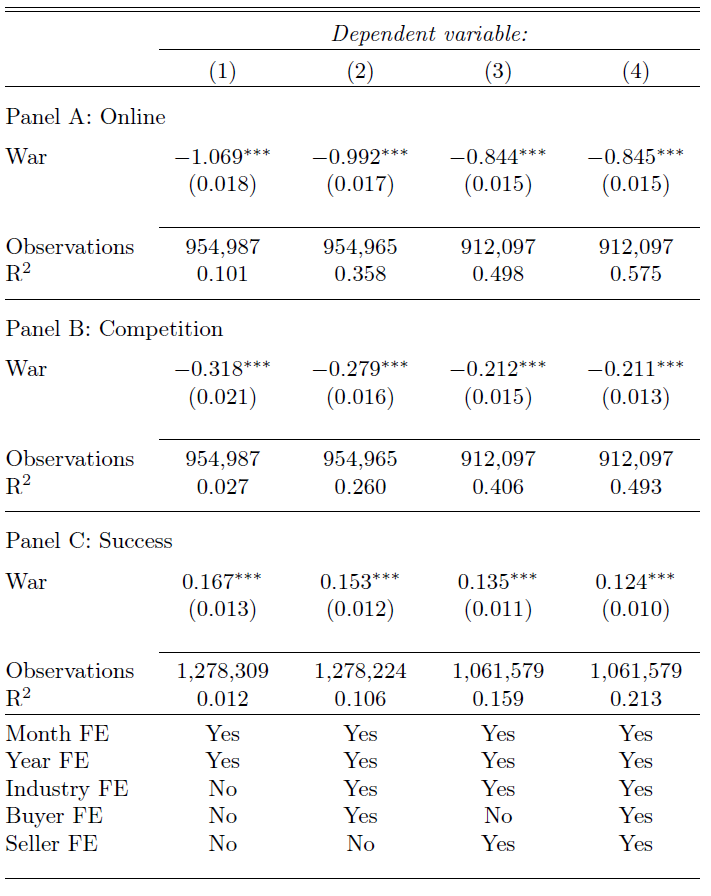

Supplement: S7 Table — Standard errors clustered at the buyer level are shown in brackets; *p < 0.1; **p < 0.05; ***p < 0.01. Each panel presents results from OLS regressions for a different dependent variable, while each column presents the results when changing the fixed effect structures adopted. All dependent variables have been standardized. (PNG) [file pone.0305344.s007.png]

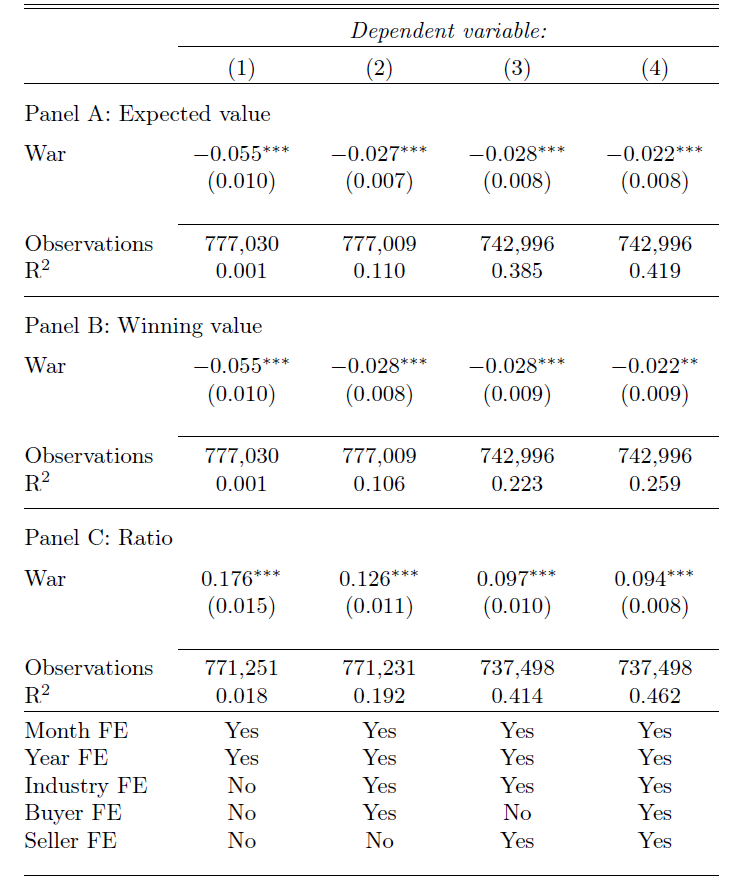

Supplement: S8 Table — Standard errors clustered at the buyer level are shown in brackets; *p < 0.1; **p < 0.05; ***p < 0.01. Each panel presents the results from OLS regressions for a different dependent variable, while each column depicts the results when changing the fixed effect structures adopted. All dependent variables have been standardized. (PNG) [file pone.0305344.s008.png]

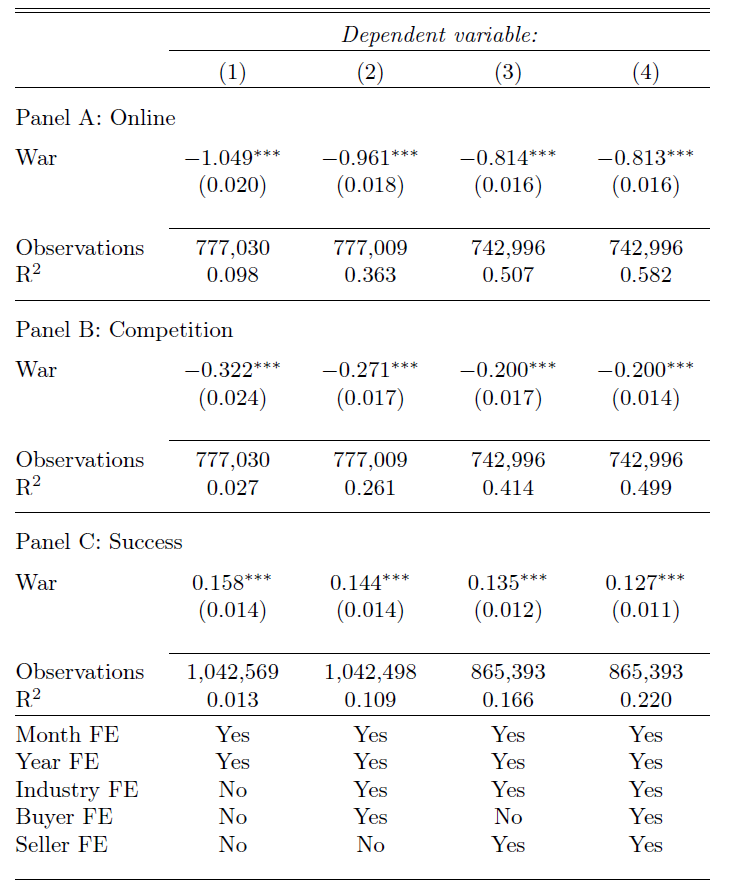

Supplement: S9 Table — Standard errors clustered at the buyer level are shown in brackets; *p < 0.1; **p < 0.05; ***p < 0.01. Each panel presents the results from OLS regressions for a different dependent variable, while each column presents the results when changing the fixed effect structures adopted. All dependent variables have been standardized. (PNG) [file pone.0305344.s009.png]

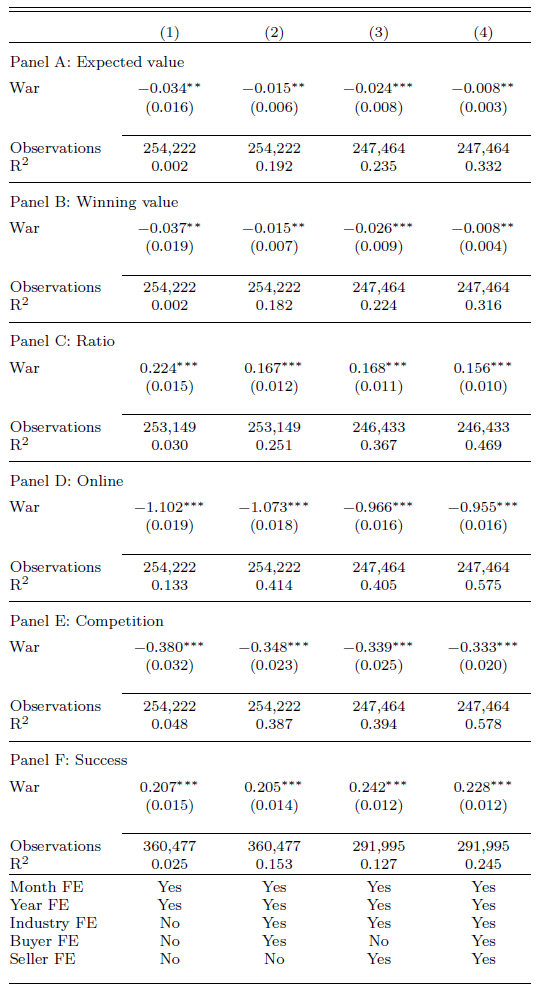

Supplement: S10 Table — Standard errors clustered at the buyer level are shown in brackets; *p < 0.1; **p < 0.05; ***p < 0.01. Each panel presents the results from OLS regressions for a different dependent variable, while each column presents the results when changing the fixed effect structures adopted. All dependent variables have been standardized. (PNG) [file pone.0305344.s010.png]

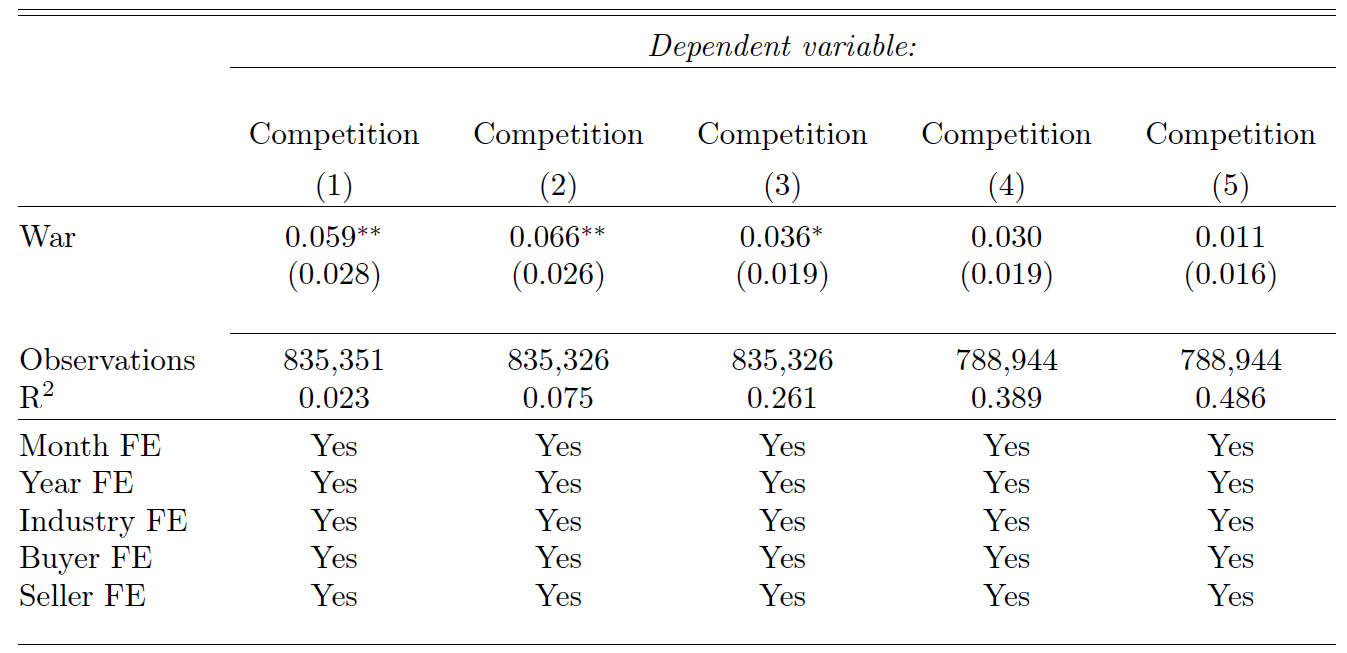

Supplement: S11 Table — Standard errors clustered at the buyer level are shown in brackets; *p < 0.1; **p < 0.05; ***p < 0.01. Each column presents the results from OLS regressions for a different dependent variable while restricting the sample to online auctions only. Full fixed effects are included. All dependent variables have been standardized. (PNG) [file pone.0305344.s011.png]

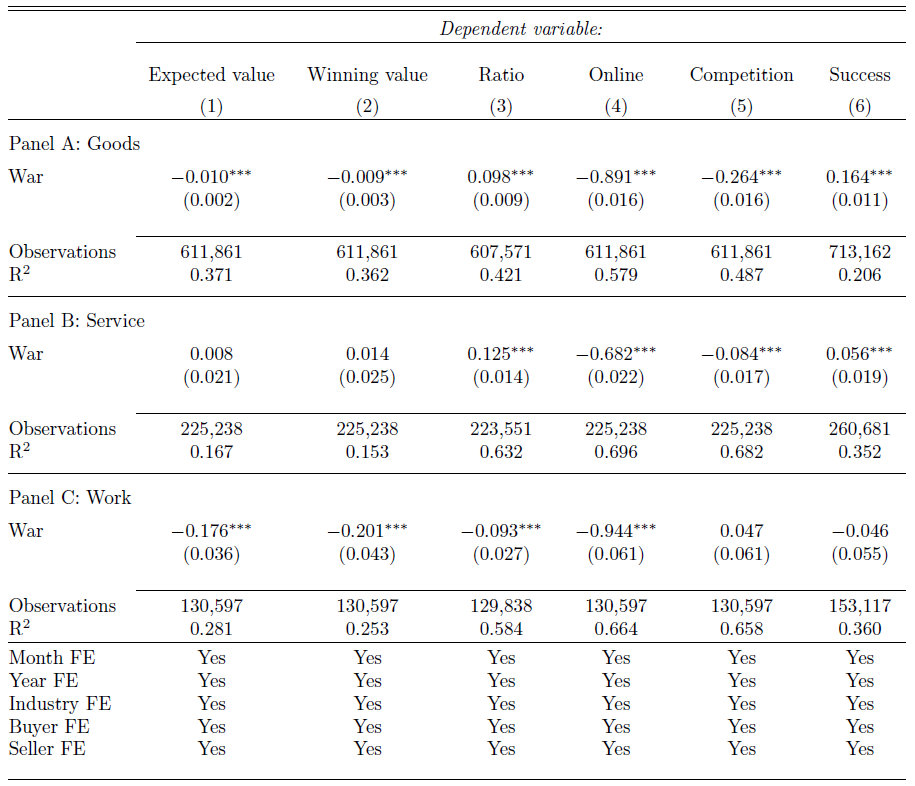

Supplement: S12 Table — Standard errors clustered at the buyer level are shown in brackets; *p < 0.1; **p < 0.05; ***p < 0.01. Each column presents the results from OLS regressions for a different dependent variable, while each panel captures a different sample restriction for goods, services, and work, respectively. All regressions included full fixed effects. All dependent variables have been standardized. (PNG) [file pone.0305344.s012.png]

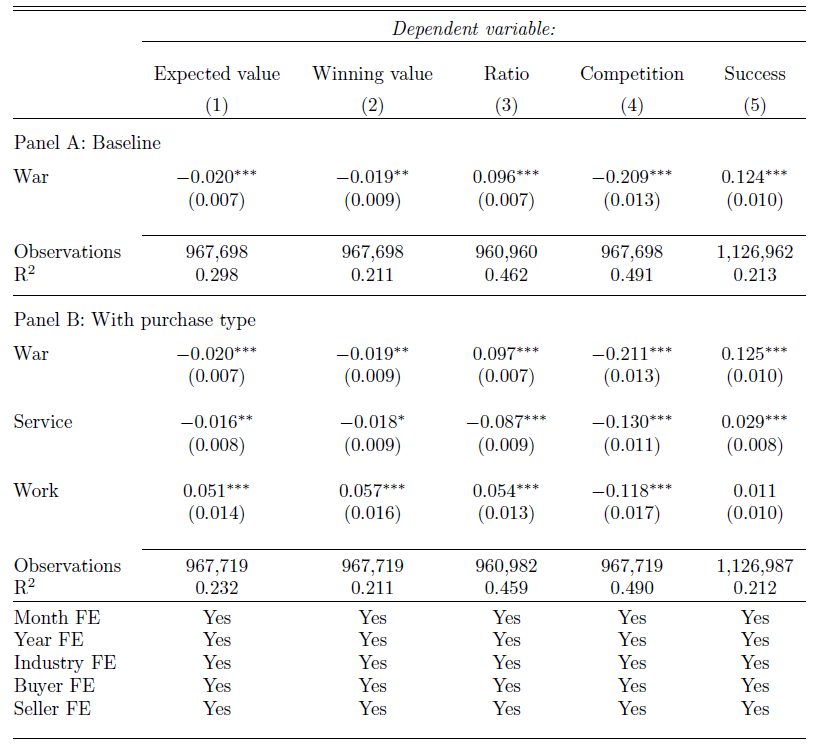

Supplement: S13 Table — Standard errors clustered at the buyer level are shown in brackets; *p < 0.1; **p < 0.05; ***p < 0.01. Each column presents the results from OLS regressions for a different dependent variable. The top panel reproduces our baseline result while the bottom panel controls for type of purchase. Full fixed effects are included. All dependent variables have been standardized. (PNG) [file pone.0305344.s013.png]

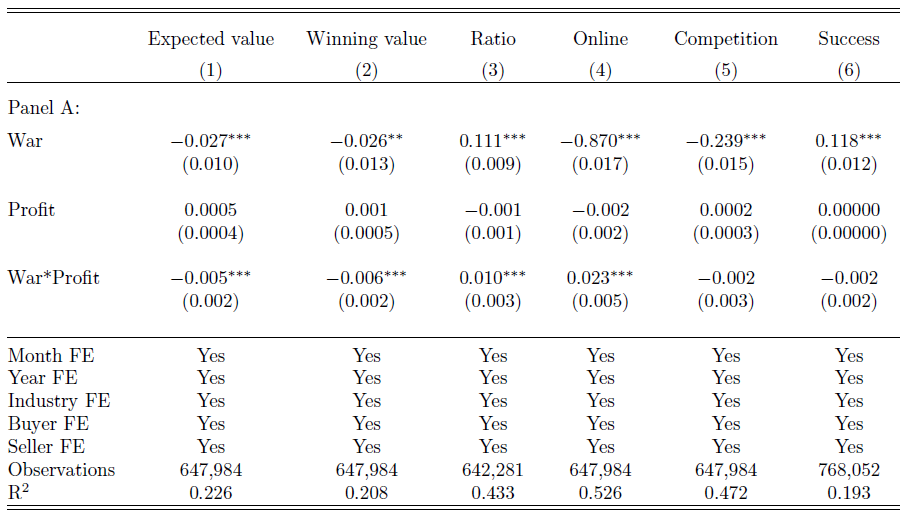

Supplement: S14 Table — Standard errors clustered at the buyer level are shown in brackets; *p < 0.1; **p < 0.05; ***p < 0.01. Each column presents the results from OLS regressions for a different dependent variable. We further include an interaction term between our dummy variable war and a measure of firm profits collected by YouControl. Full fixed effects are included. All dependent variables have been standardized. (PNG) [file pone.0305344.s014.png]

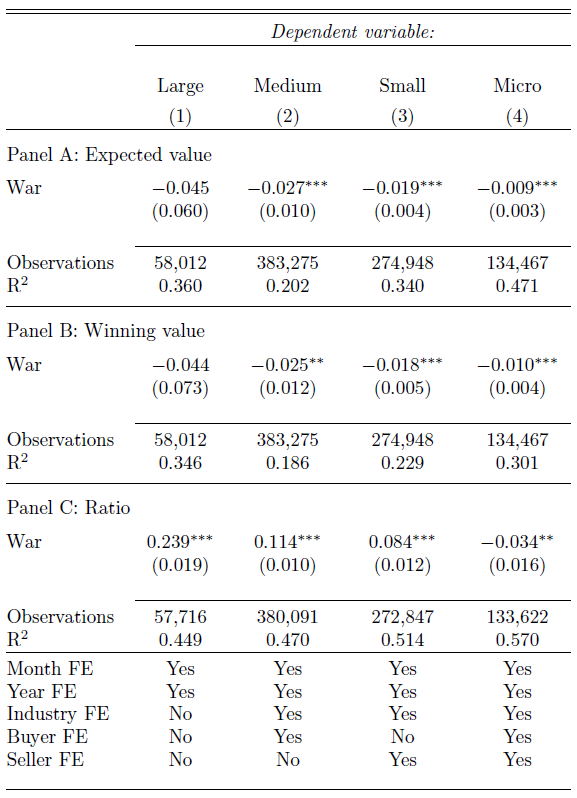

Supplement: S15 Table — Standard errors clustered at the buyer level are shown in brackets; *p < 0.1; **p < 0.05; ***p < 0.01. Each panel presents the results from OLS regressions for a different dependent variable, while each column presents results when changing the fixed effect structures adopted. All dependent variables have been standardized. (PNG) [file pone.0305344.s015.png]

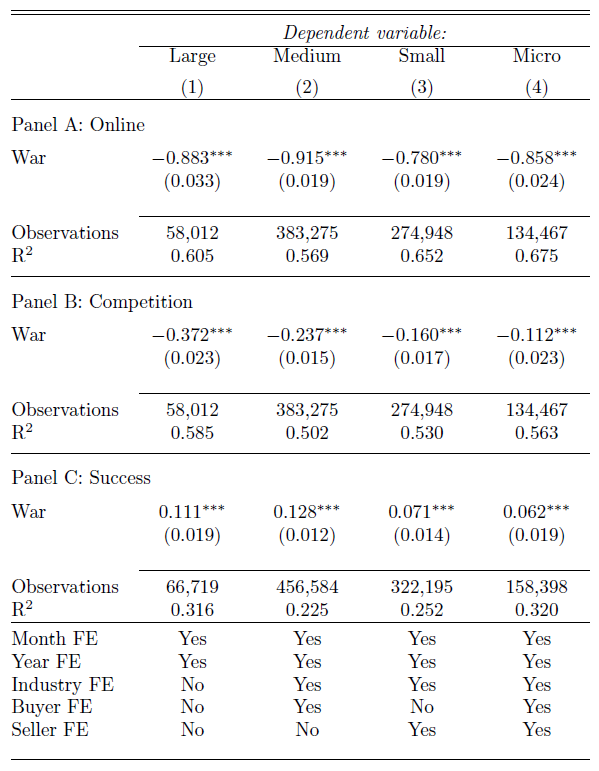

Supplement: S16 Table — Standard errors clustered at the buyer level are shown in brackets; *p < 0.1; **p < 0.05; ***p < 0.01. Each panel presents the results from OLS regressions for a different dependent variable, while each column presents the results when changing the fixed effect structures adopted. All dependent variables have been standardized. (PNG) [file pone.0305344.s016.png]

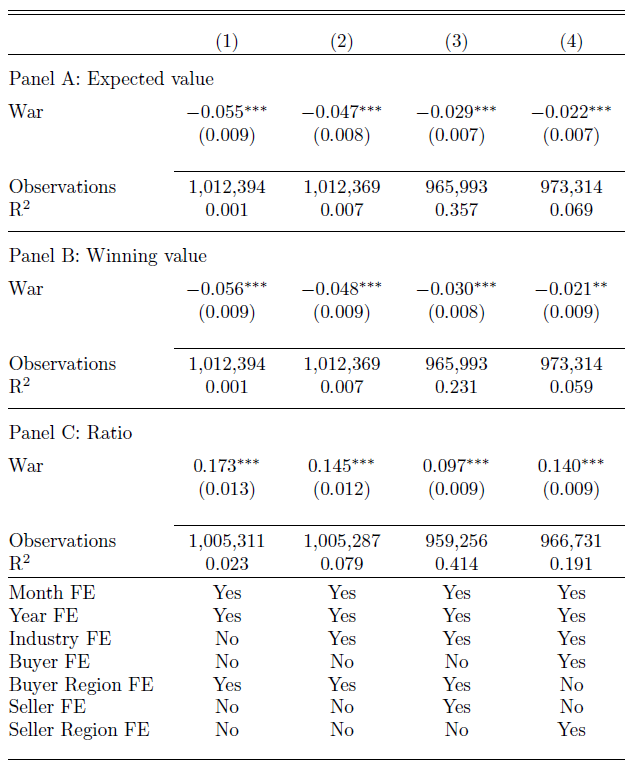

Supplement: S17 Table — Standard errors clustered at the buyer level are shown in brackets; *p < 0.1; **p < 0.05; ***p < 0.01. Each panel presents the results from OLS regressions for a different dependent variable, while each column presents results when changing the fixed effect structures adopted. All dependent variables have been standardized. (PNG) [file pone.0305344.s017.png]

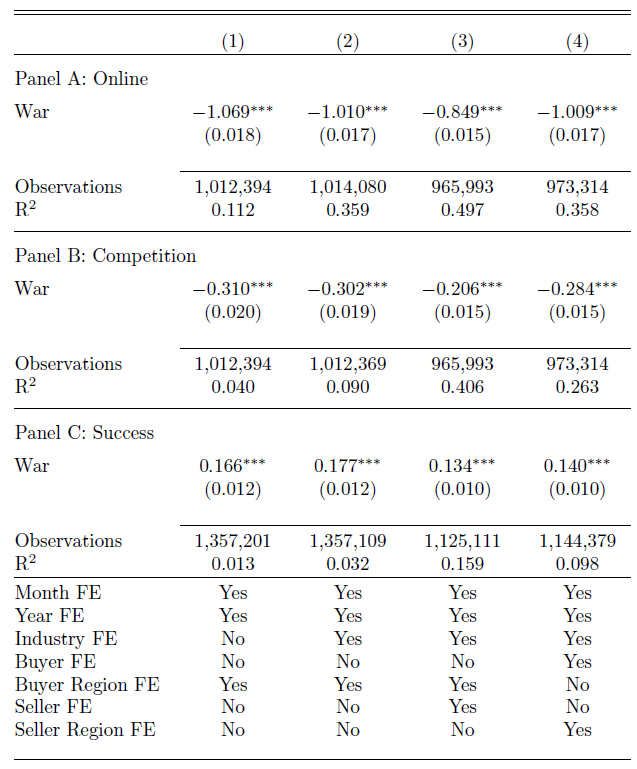

Supplement: S18 Table — Standard errors clustered at the buyer level are shown in brackets; *p < 0.1; **p < 0.05; ***p < 0.01. Each panel presents the results from OLS regressions for a different dependent variable, while each column presents the results when changing the fixed effect structures adopted. All dependent variables have been standardized. (PNG) [file pone.0305344.s018.png]

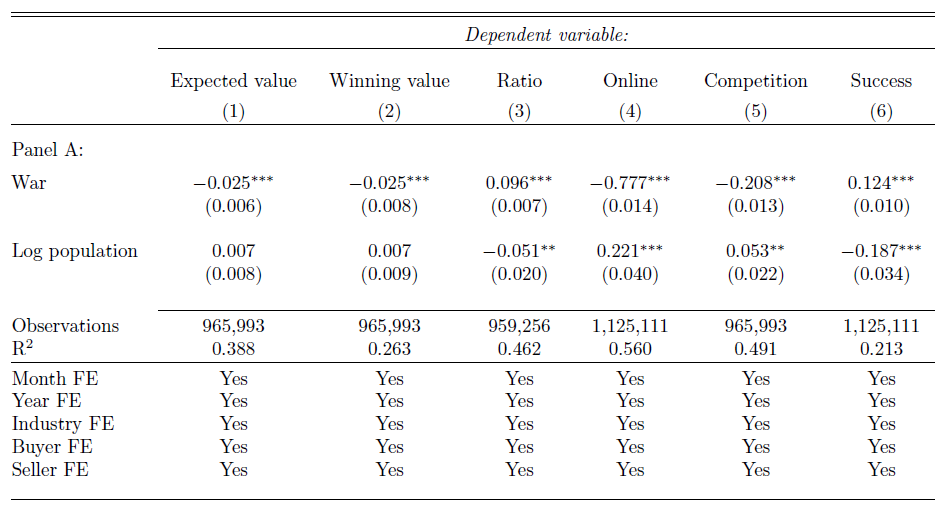

Supplement: S19 Table — Standard errors clustered at the buyer level are shown in brackets; *p < 0.1; **p < 0.05; ***p < 0.01. Each panel presents the results from OLS regressions for a different dependent variable, while each column presents the results when changing the fixed effect structures adopted. All dependent variables have been standardized. This table controls for region population to account for the potential non-linear scaling of procurement activity with population size. (PNG) [file pone.0305344.s019.png]

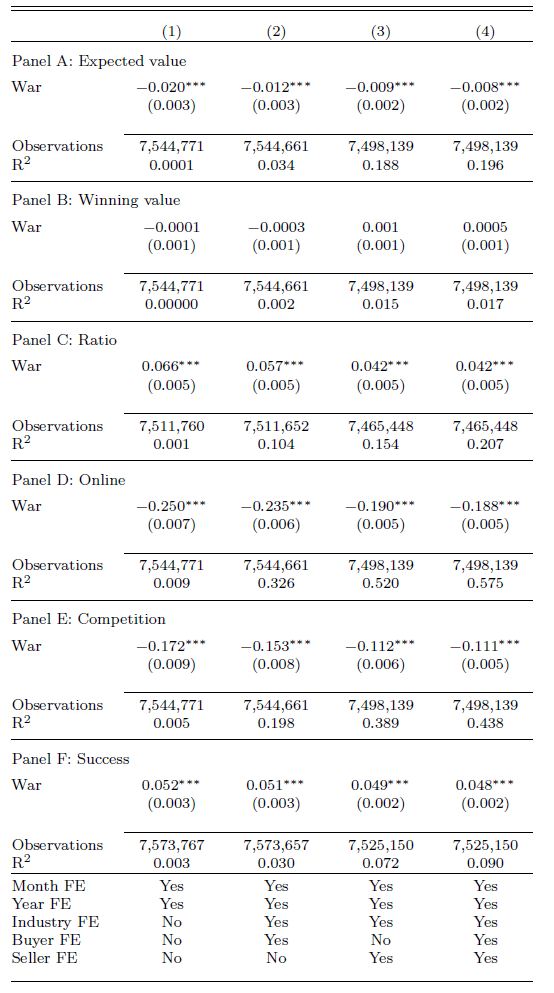

Supplement: S20 Table — Standard errors clustered at the buyer level are shown in brackets; *p < 0.1; **p < 0.05; ***p < 0.01. Each panel presents the results from OLS regressions for a different dependent variable, while each column presents the results when changing the fixed effect structures adopted. All dependent variables have been standardized. The sample includes purchases of any value. (PNG) [file pone.0305344.s020.png]

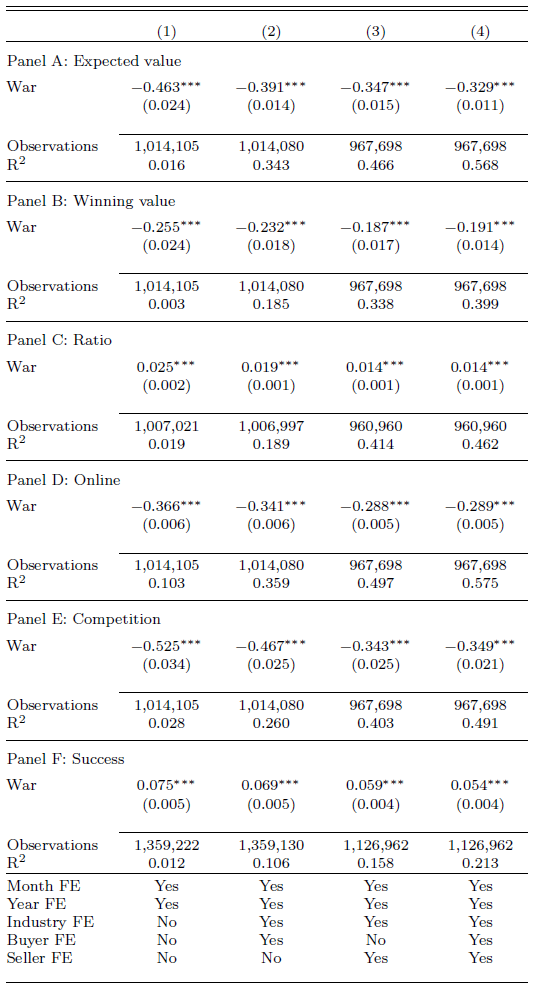

Supplement: S21 Table — Standard errors clustered at the buyer level are shown in brackets; *p < 0.1; **p < 0.05; ***p < 0.01. Each panel presents the results from OLS regressions for a different dependent variable, while each column presents the results when changing the fixed effect structures adopted. (PNG) [file pone.0305344.s021.png]

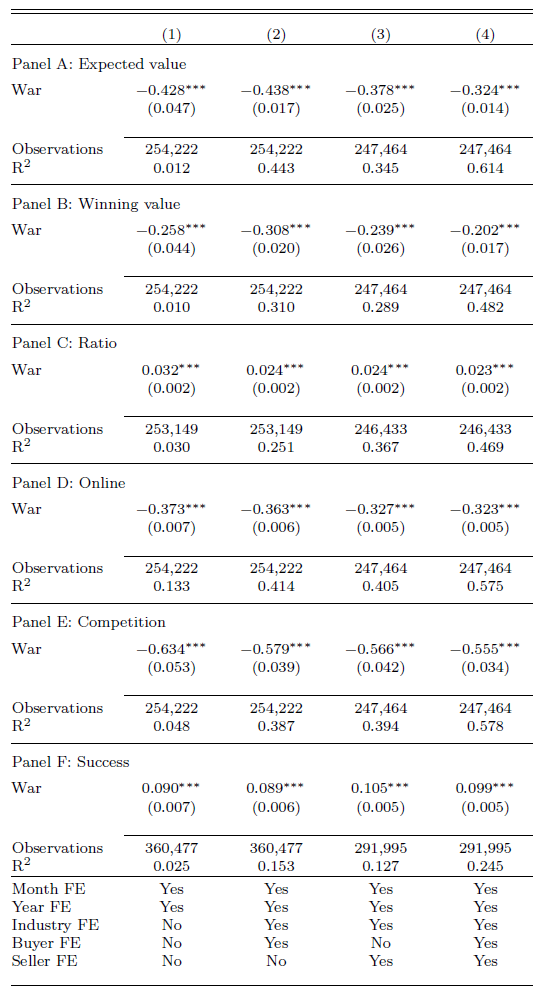

Supplement: S22 Table — Standard errors clustered at the buyer level are shown in brackets; *p < 0.1; **p < 0.05; ***p < 0.01. Each panel presents the results from OLS regressions for a different dependent variable, while each column presents the results when changing the fixed effect structures adopted. (PNG) [file pone.0305344.s022.png]

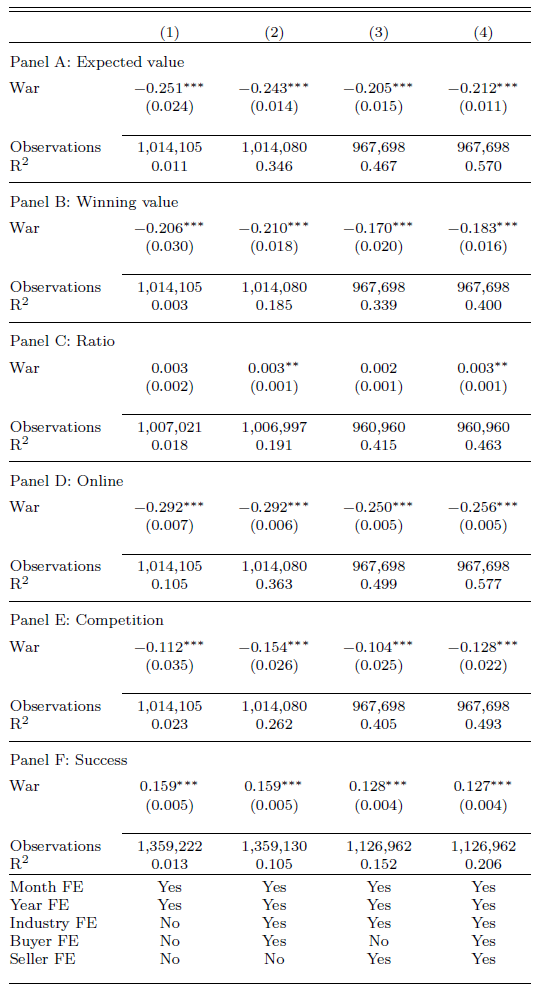

Supplement: S23 Table — Standard errors clustered at the buyer level are shown in brackets; *p < 0.1; **p < 0.05; ***p < 0.01. Each panel presents results from the OLS regressions for a different dependent variable, while each column depicts the results when changing the fixed effect structures adopted. Every procurement transaction has two reference dates: the tender date captured when the purchase occurs; and the publishing date referring to the time the buyer posted the call for tender. Our baseline regressions throughout the analyses use the tender dates as the reference time, and we reproduce our analyses with the publication date in this regression. (PNG) [file pone.0305344.s023.png]

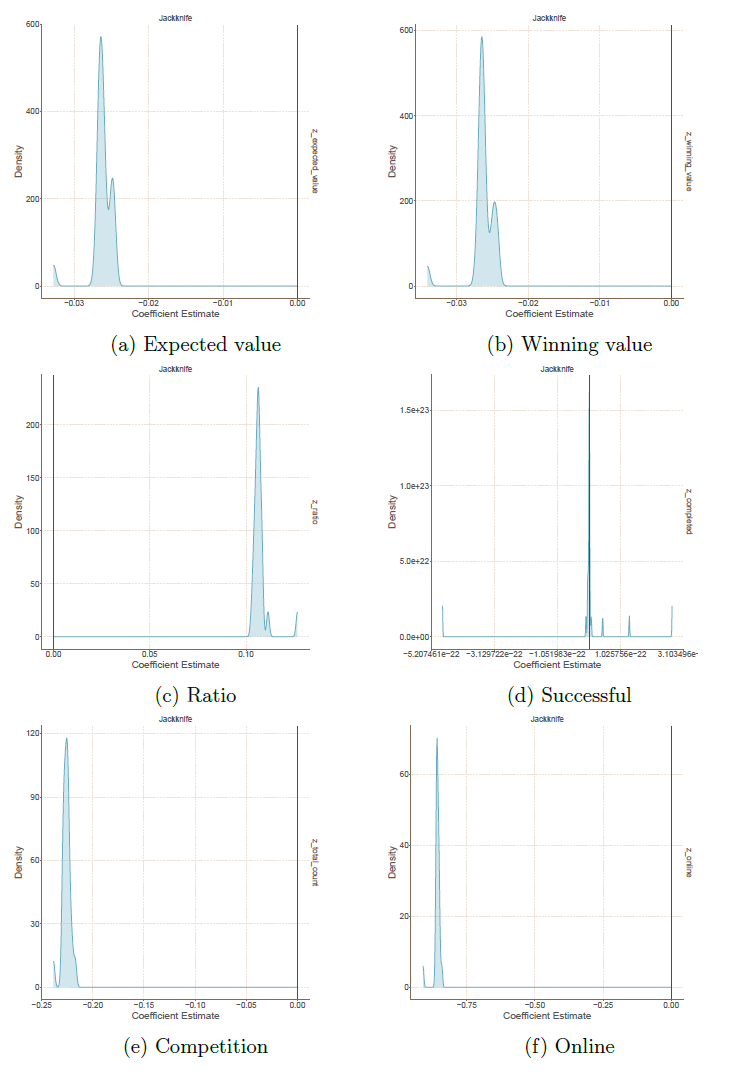

Supplement: S1 Fig — Jackknife test results for regions in Ukraine. We drop each of the 24 Ukraine regions in turn to determine the coefficients. The pre-was period is from January 2021 until February 2022, while the post-war period is from 24 February 2022 until the end of October 2022. (PNG) [file pone.0305344.s024.png]

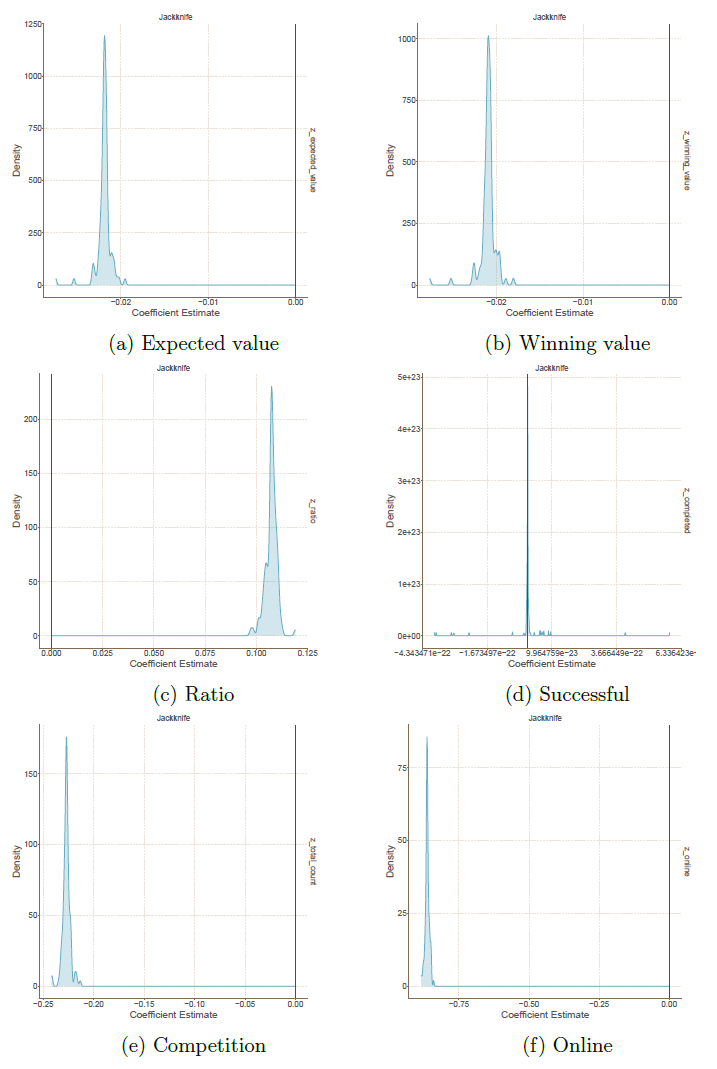

Supplement: S2 Fig — Jackknife test results for weeks. We drop each week in each year in our dataset in turn to determine the coefficients. The pre-was period is from January 2021 until February 2022, while the post-war period is from 24 February 2022 until the end of October 2022. (PNG) [file pone.0305344.s025.png]

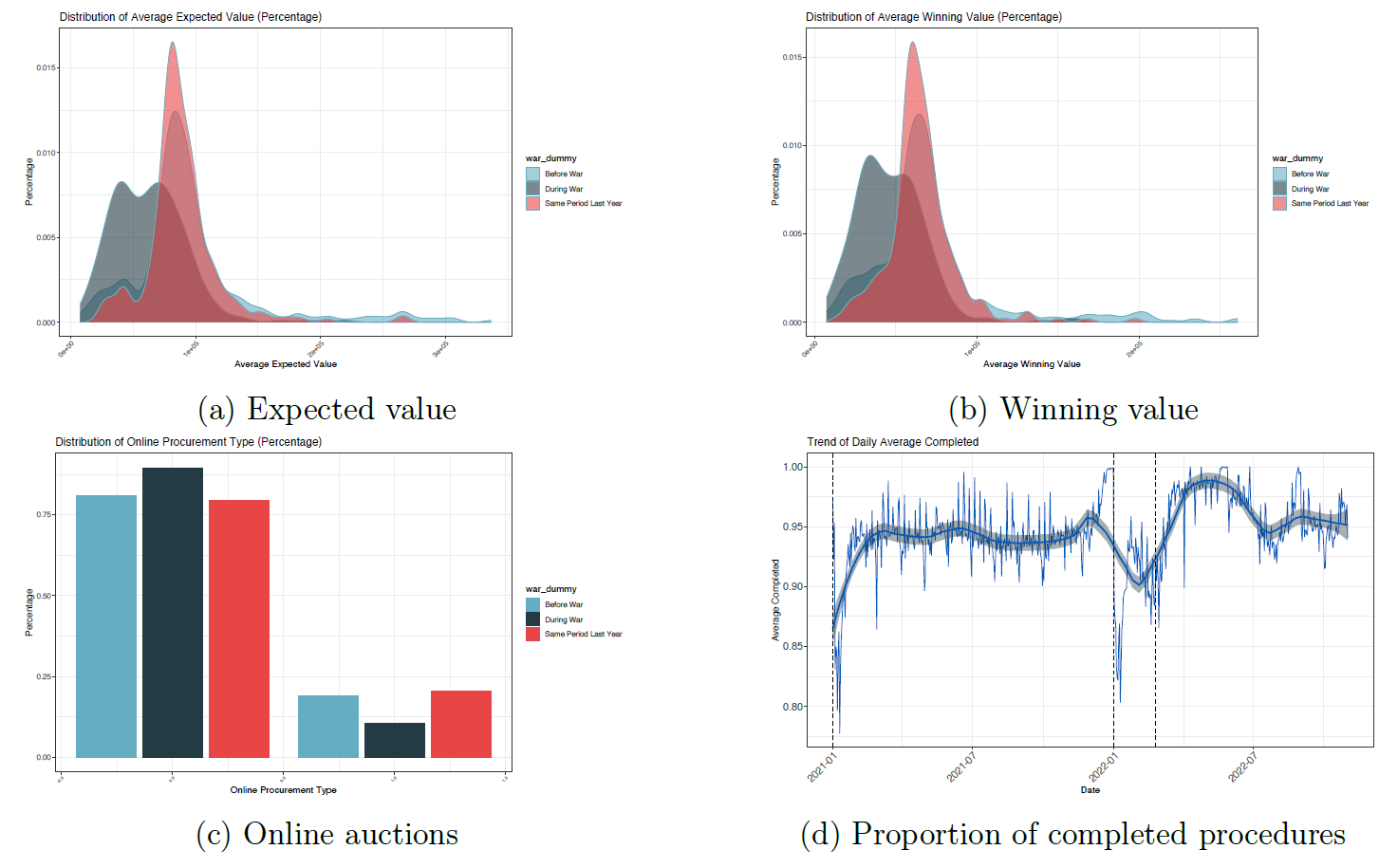

Supplement: S3 Fig — (PNG) [file pone.0305344.s026.png]

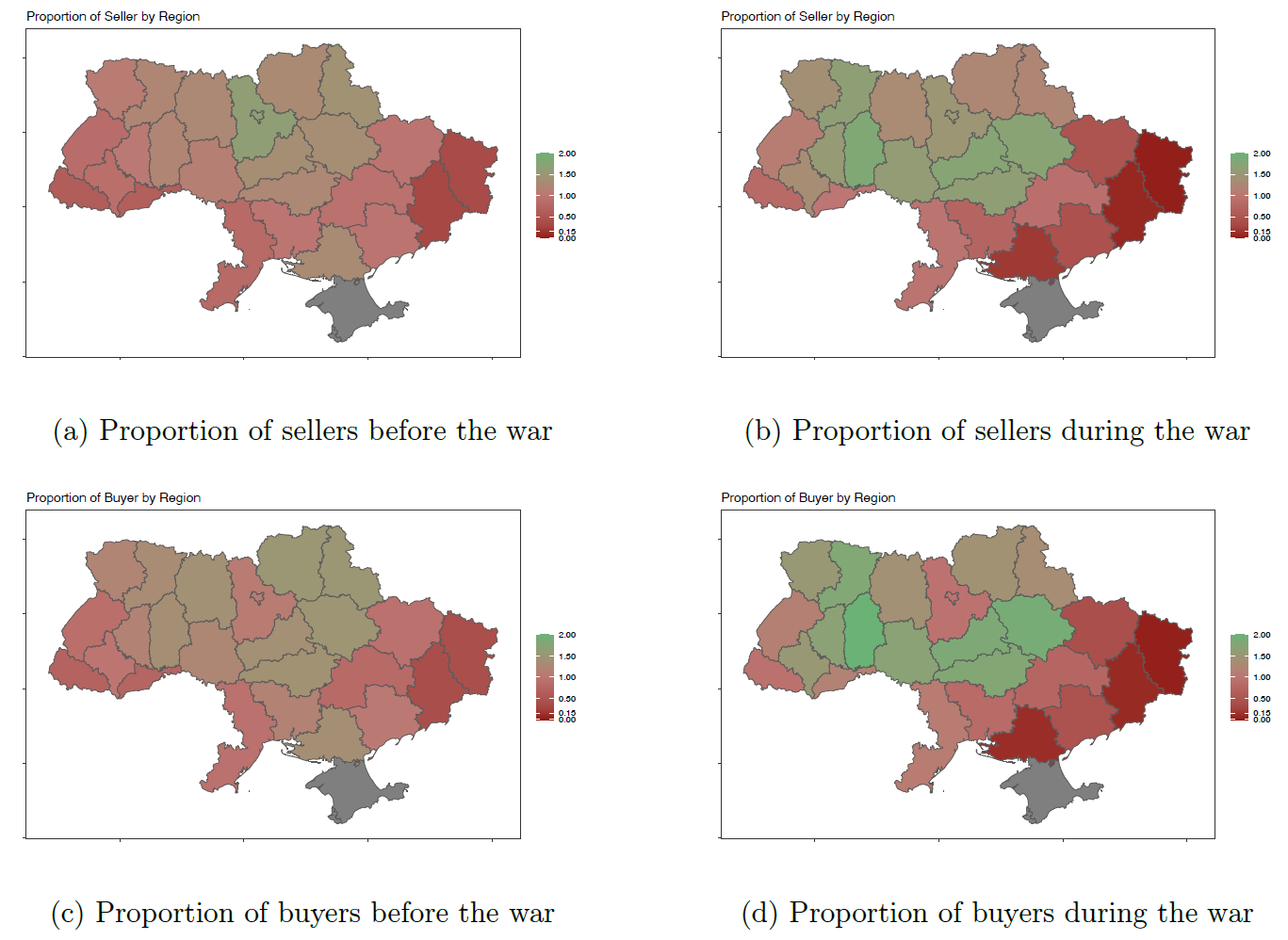

Supplement: S4 Fig — Proportion of sellers and buyers in each region of Ukraine before and during the war, weighed by the population proportion in each of these regions, respectively. The pre-was period is from January 2021 until February 2022, while the post-war period is from 24 February 2022 until the end of October 2022. (PNG) [file pone.0305344.s027.png]

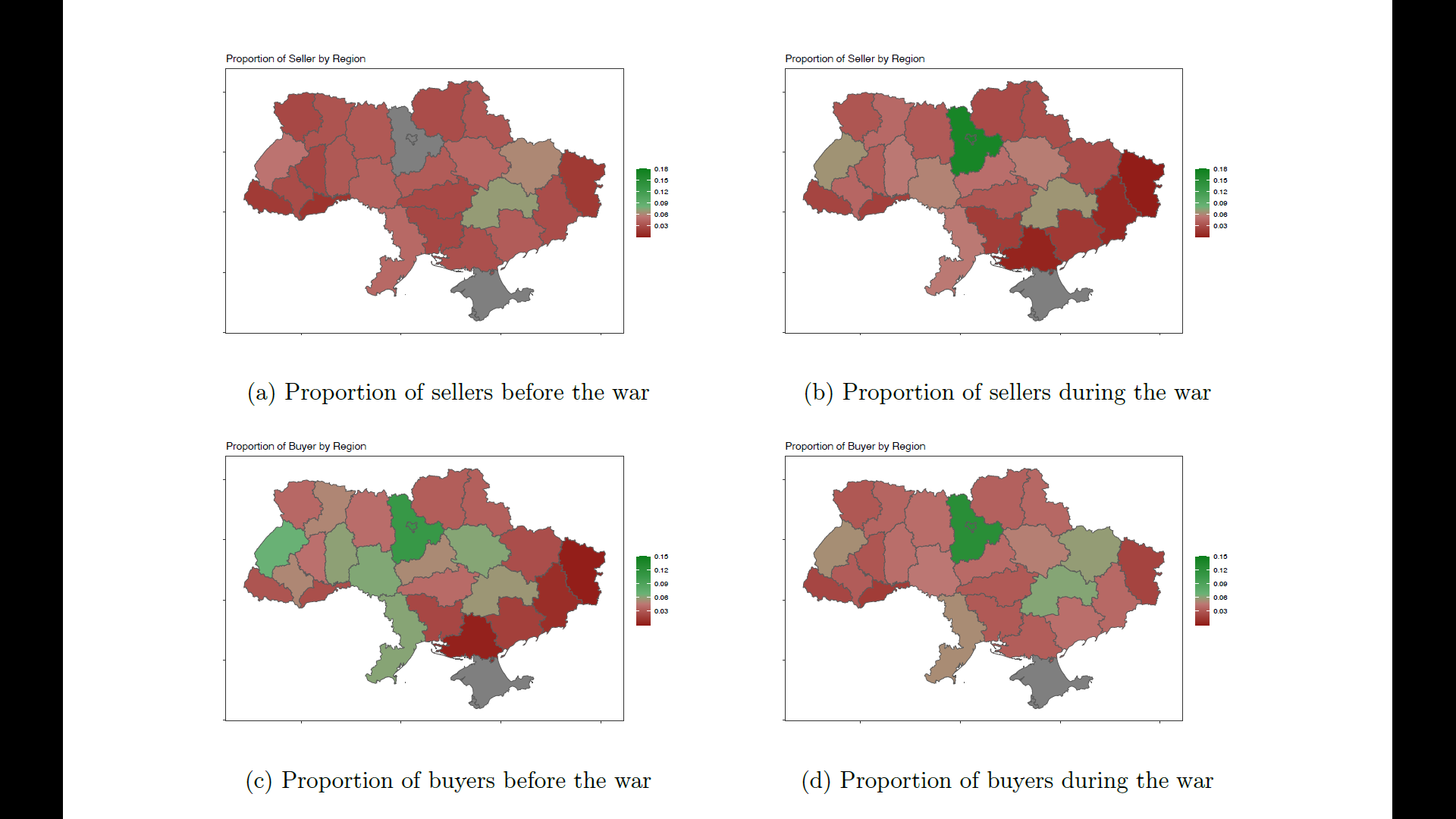

Supplement: S5 Fig — Proportion of sellers and buyers in each region of Ukraine before and during the war. The pre-was period is from January 2021 until February 2022, while the post-war period is from 24 February 2022 until the end of October 2022. (PNG) [file pone.0305344.s028.png]

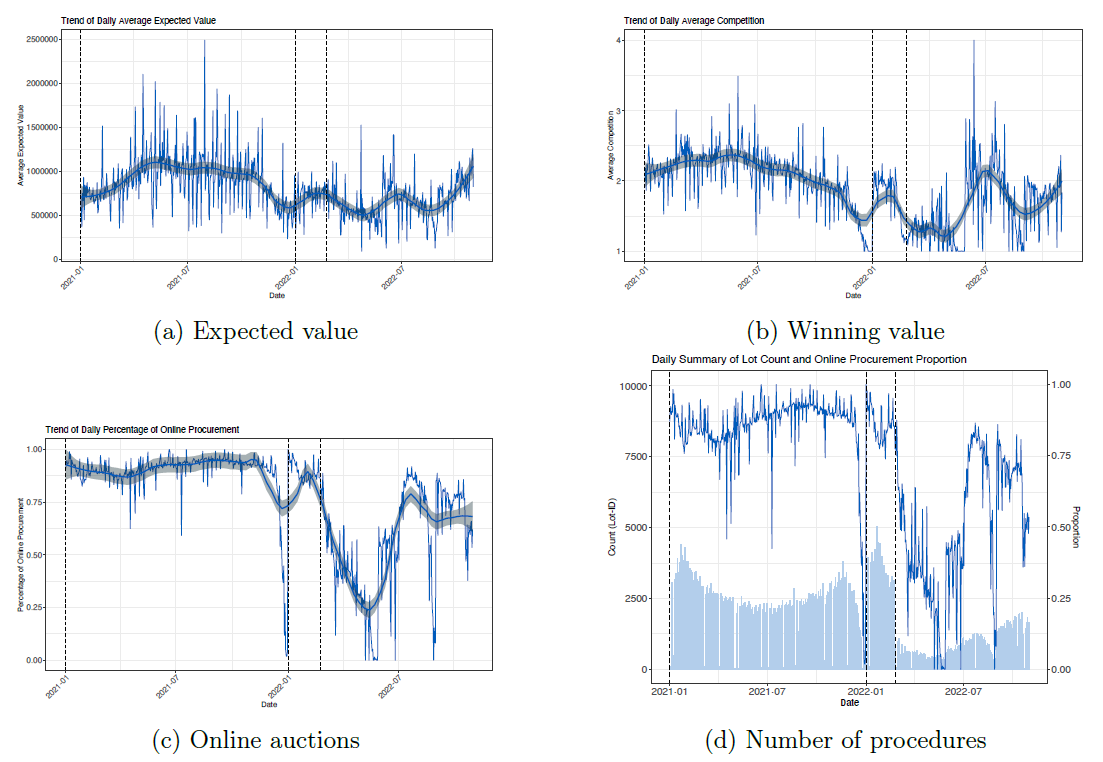

Supplement: S6 Fig — Proportion of sellers and buyers in each region of Ukraine before and during the war weighted by population. The pre-was period is from January 2021 until February 2022, while the post-war period is from 24 February 2022 until the end of October 2022. The black and vertical lines indicate the beginning of the year and the invasion, respectively. (PNG) [file pone.0305344.s029.png]
