# Supplementary material for: Governance in times of war: Public procurement in Ukraine
Source: PLoS One. 2024 Jun 21;19(6):e0305344. doi: 10.1371/journal.pone.0305344 (PMC11192321; doi:10.1371/journal.pone.0305344)
Supplement: S1 File — S1 Appendix. Results with standardized dependent variables. S1.1 Jackknife results. S2 Appendix. Results with non-standardized dependent variables. S2.1 Tender date. S2.2 Publishing date. S3 Appendix. Other results. S4 Appendix. Results for procurement above 50,000. S5 Appendix. Procurement system. (DOCX) [file pone.0305344.s030.docx]

# Supporting Information

# Governance in times of War:

# Public procurement in Ukraine

Margaryta Klymak^1¶^, Tim Vlandas^2¶^∗

^1^ Department of International Development, Kings College London, United Kingdom

^2^ Department of Social Policy and Intervention, University of Oxford, United Kingdom

^*^ Corresponding author

E-mail: [tim.vlandas@spi.ox.ac.uk](mailto:tim.vlandas@spi.ox.ac.uk)

^¶^These authors contributed equally to this work.

# S1 Appendix . Results with standardized dependent variables

### **S1 Table. Effect of the war—full results plotted in Fig 3.**


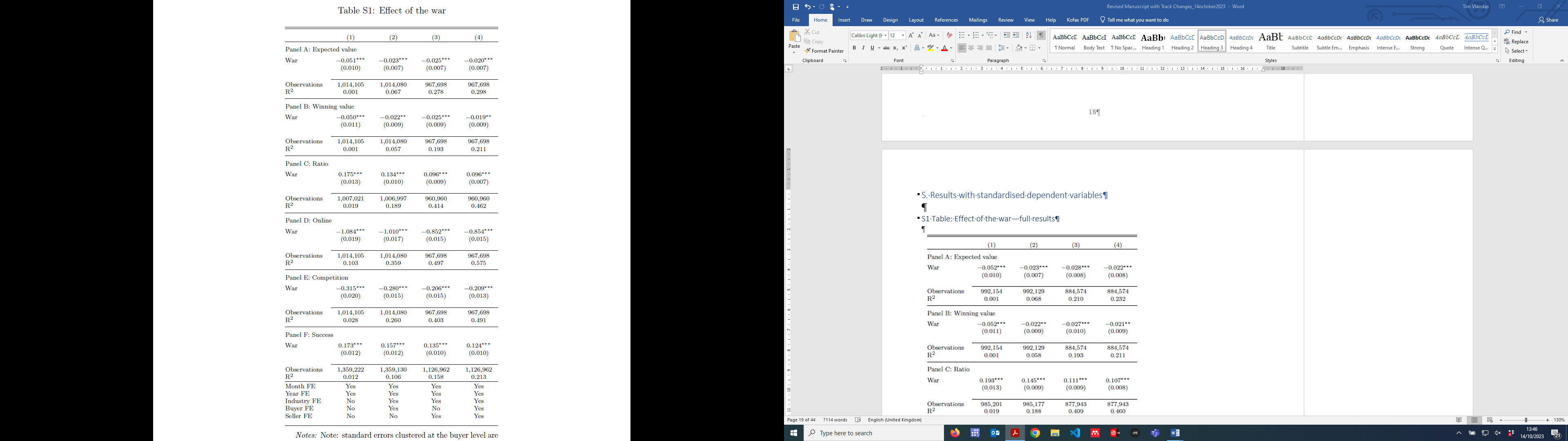


Standard errors clustered at the buyer level are shown in brackets; ∗p < 0.1; ∗∗p < 0.05; ∗∗∗p < 0.01. Clustering at the seller level does not change our conclusions. Each panel presents the results from ordinary least squares (OLS) regressions for a different dependent variable, while each column presents the results when changing the fixed effect structures adopted. All dependent variables have been standardized.

### **S2 Table. Effect of the war when controlling for policy changes (1).**


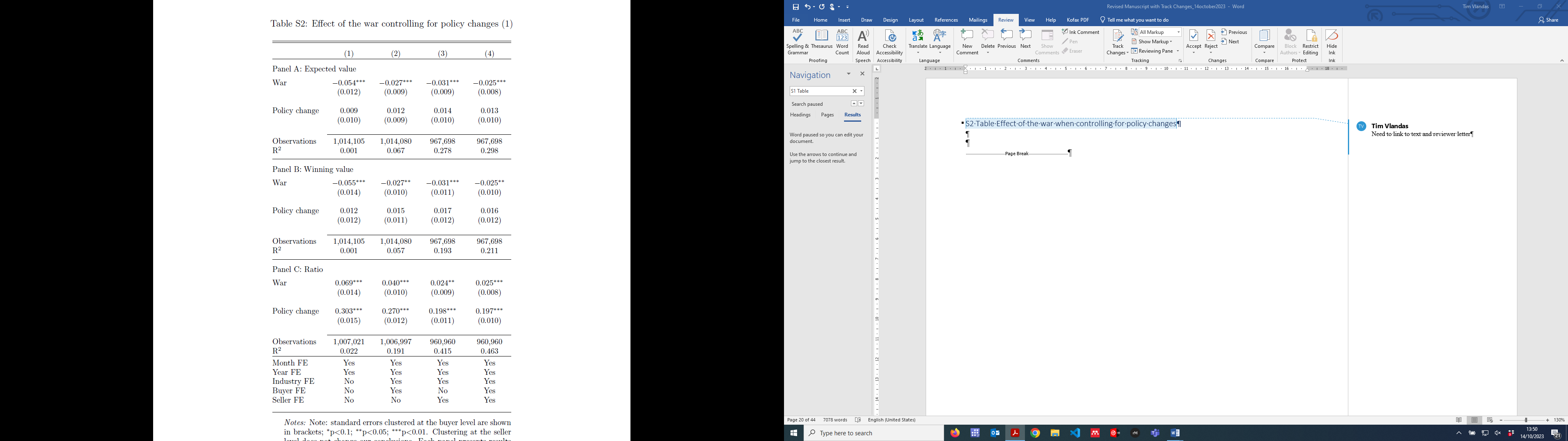


Standard errors clustered at the buyer level are shown in brackets; ∗p < 0.1; ∗∗p < 0.05; ∗∗∗p < 0.01. Each panel presents the results from OLS regressions for a different dependent variable, while each column presents the results when changing the fixed effect structures adopted. All dependent variables have been standardized. S5 Appendix provides more information about the policy change.

### **S3 Table. Effect of the war when controlling for policy changes (2).**


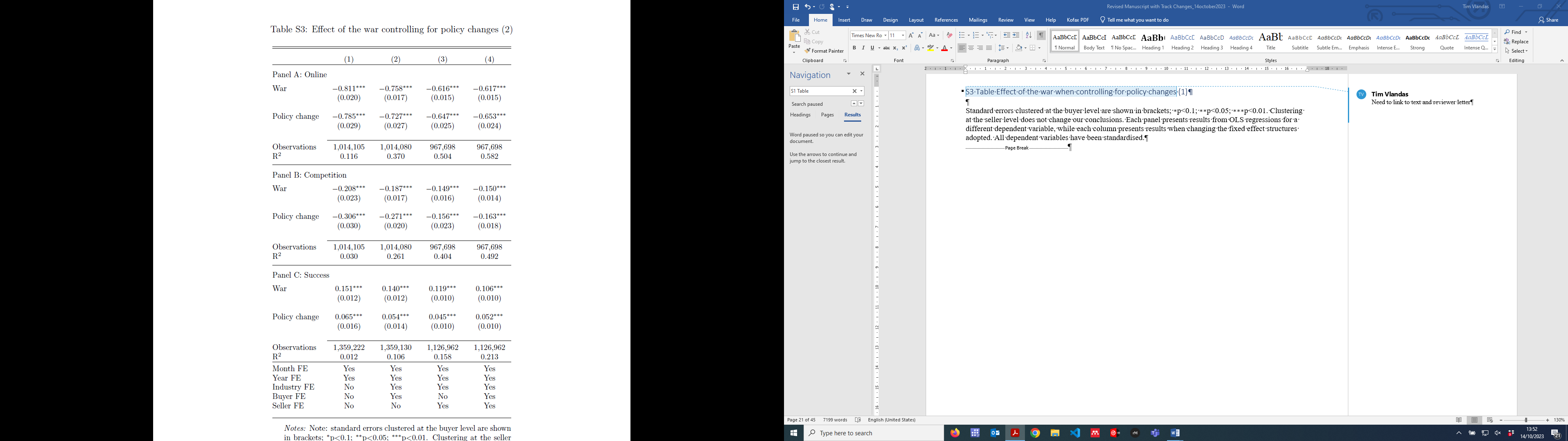


Standard errors clustered at the buyer level are shown in brackets; ∗p < 0.1; ∗∗p < 0.05; ∗∗∗p < 0.01. Each panel presents the results from OLS regressions for a different dependent variable, while each column presents the results when changing the fixed effect structures adopted. All dependent variables have been standardized. S5 Appendix presents more information about the policy change.

### **S4 Table. Effect of the war during the early and late war stages.**


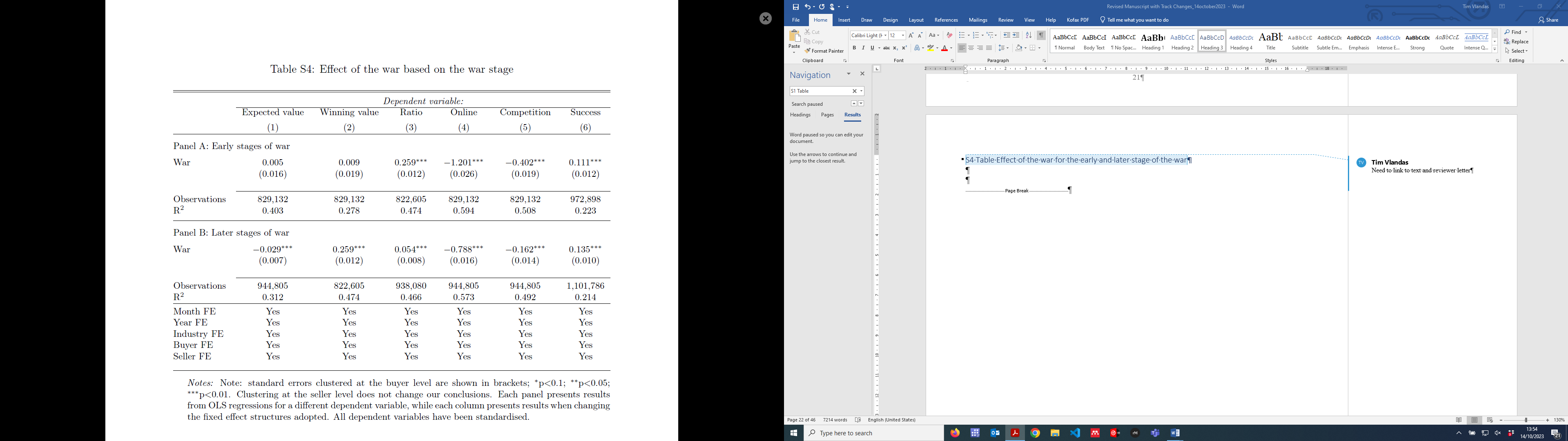


Standard errors clustered at the buyer level are shown in brackets; ∗p < 0.1; ∗∗p < 0.05; ∗∗∗p < 0.01. Each column presents the results from OLS regressions for a different dependent variable. The top panel restricts the sample to the early stages of the war (first two months), while the bottom panel does the same but for the later stages of the war. All columns include all fixed effects. All dependent variables have been standardized.

### **S5 Table. Effect of the war when excluding observations for the capital region.**


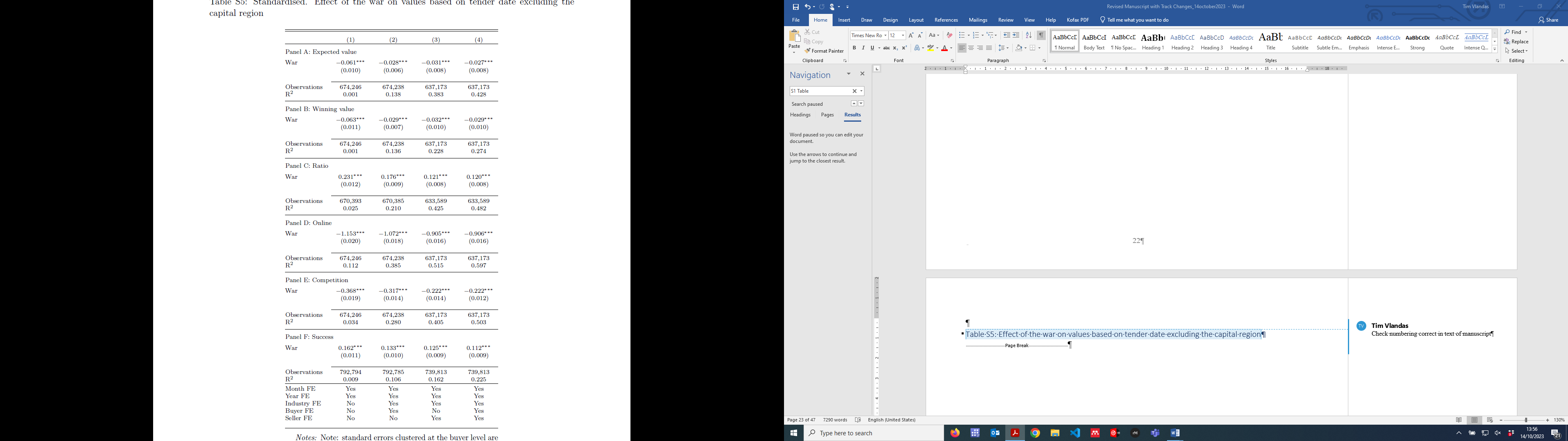


Standard errors clustered at the buyer level are shown in brackets; ∗p < 0.1; ∗∗p < 0.05; ∗∗∗p < 0.01. Each panel presents the results from OLS regressions for a different dependent variable, while each column presents the results when changing the fixed effect structures adopted. All dependent variables have been standardized.

### **S6 Table. Effect of the war when excluding observations for the Luhansk and Donetsk regions (1).**


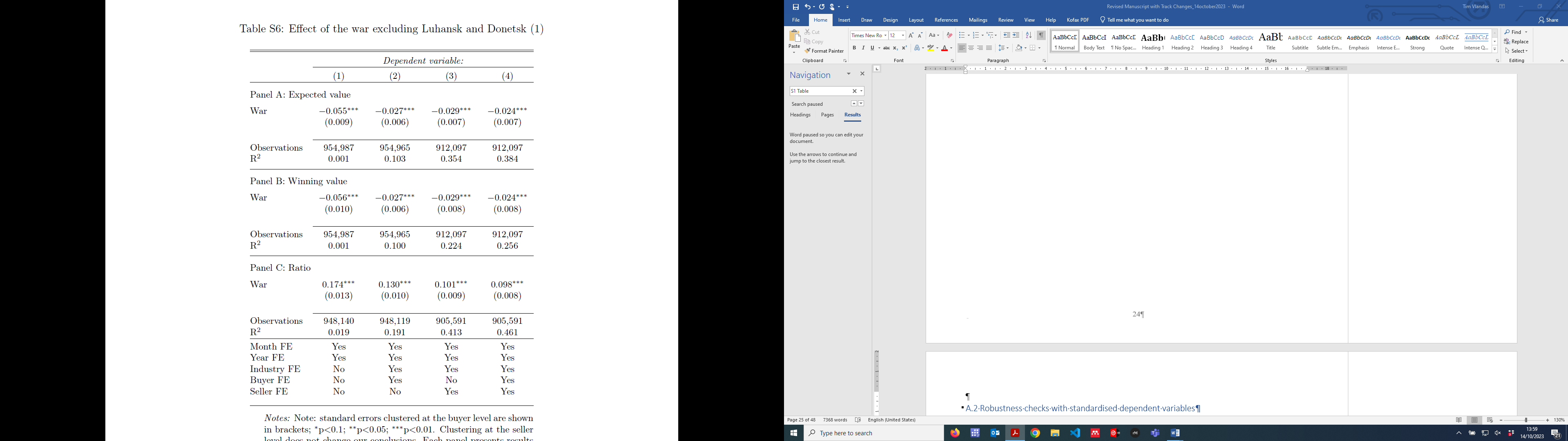


Standard errors clustered at the buyer level are shown in brackets; ∗p < 0.1; ∗∗p < 0.05; ∗∗∗p < 0.01. Each panel presents the results from OLS regressions for a different dependent variable, while each column presents the results when changing the fixed effect structures adopted. All dependent variables have been standardized.

### **S7 Table. Effect of the war when excluding observations for the Luhansk and Donetsk regions (2).**


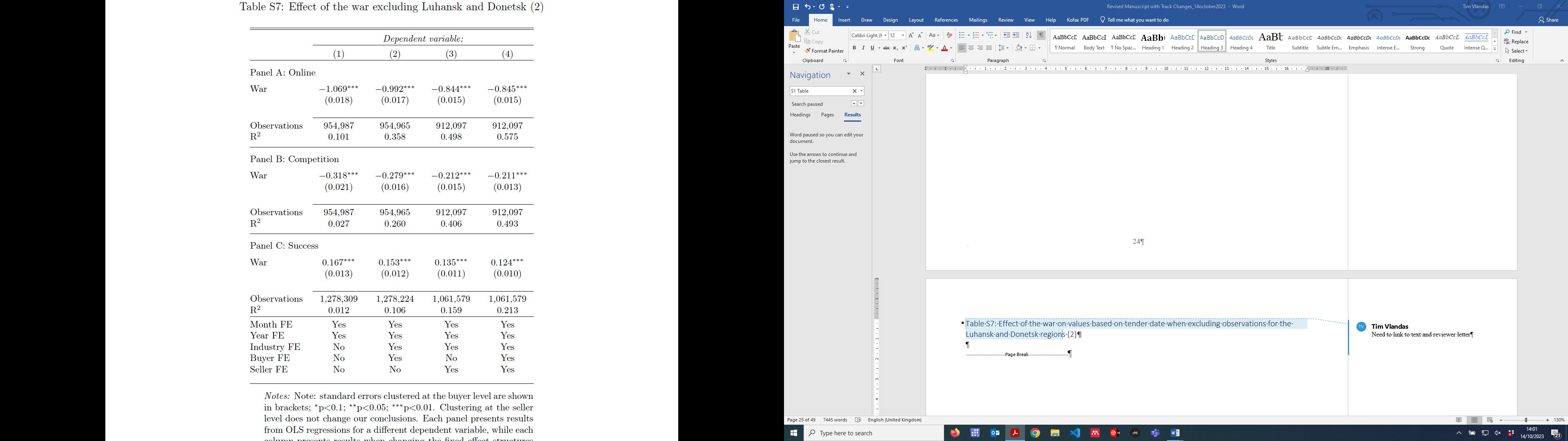


Standard errors clustered at the buyer level are shown in brackets; ∗p < 0.1; ∗∗p < 0.05; ∗∗∗p < 0.01. Each panel presents results from OLS regressions for a different dependent variable, while each column presents the results when changing the fixed effect structures adopted. All dependent variables have been standardized.

### **S8 Table . Effect of the war when excluding observations for Luhansk, Donetsk, and the neighboring regions (1).**


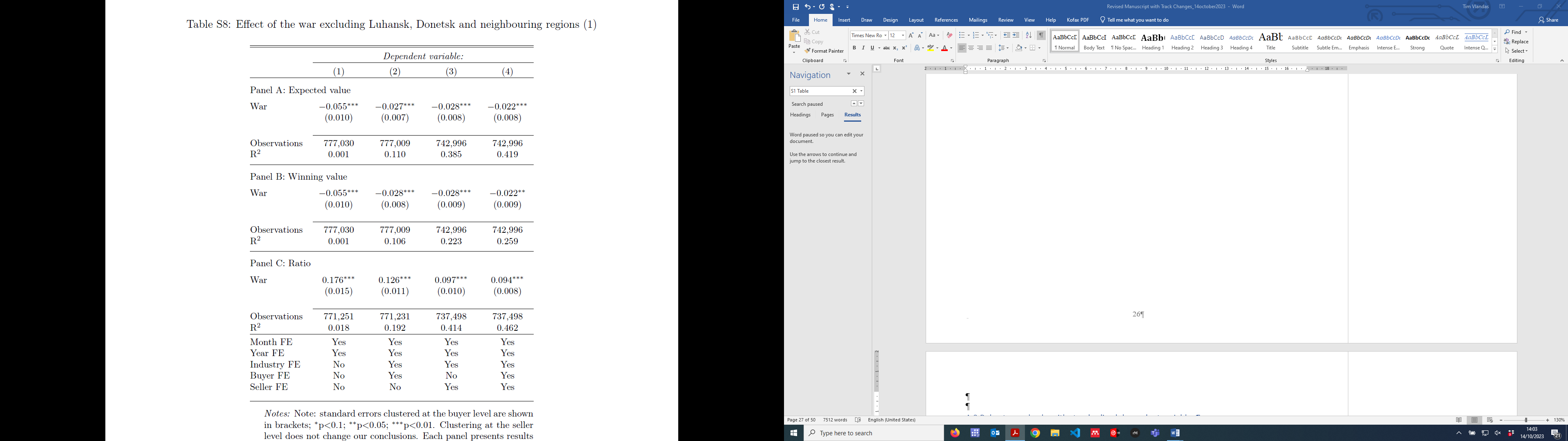


Standard errors clustered at the buyer level are shown in brackets; ∗p < 0.1; ∗∗p < 0.05; ∗∗∗p < 0.01. Each panel presents the results from OLS regressions for a different dependent variable, while each column depicts the results when changing the fixed effect structures adopted. All dependent variables have been standardized.

### **S9 Table. Effect of the war when excluding observations for Luhansk, Donetsk, and the neighboring regions (2).**


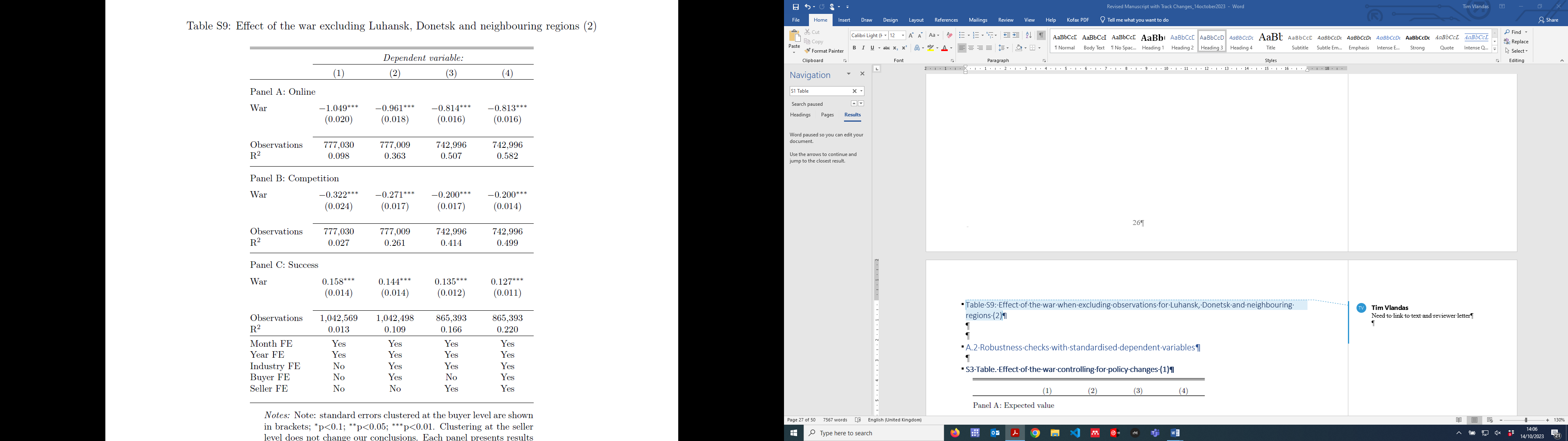


Standard errors clustered at the buyer level are shown in brackets; ∗p < 0.1; ∗∗p < 0.05; ∗∗∗p < 0.01. Each panel presents the results from OLS regressions for a different dependent variable, while each column presents the results when changing the fixed effect structures adopted. All dependent variables have been standardized.

### **S10 Table. Effect of the war restricting observations to homogenous goods only.**


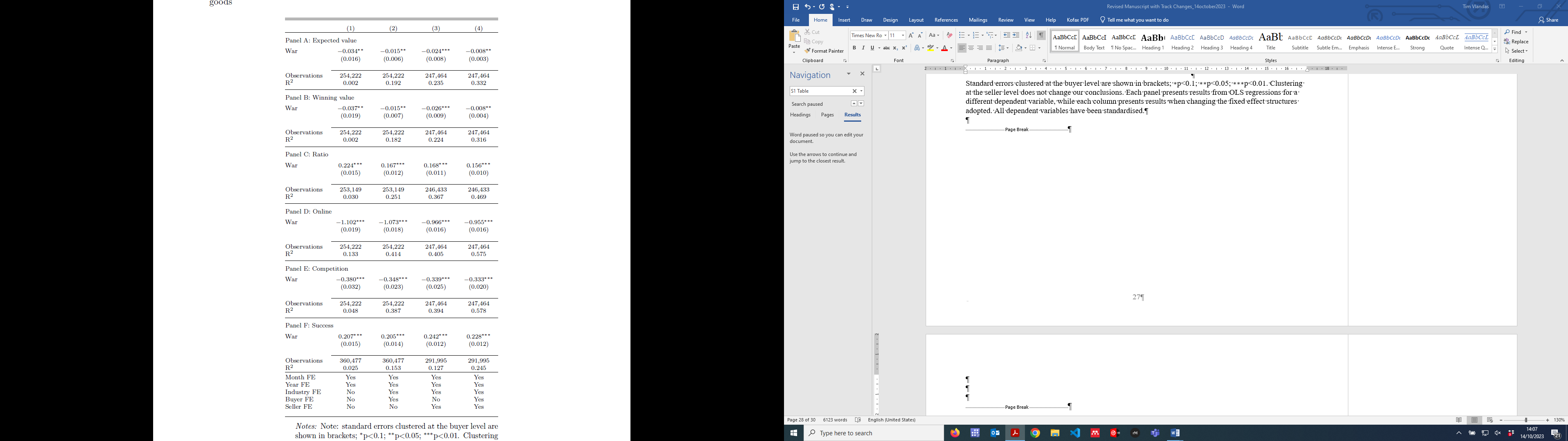


Standard errors clustered at the buyer level are shown in brackets; ∗p < 0.1; ∗∗p < 0.05; ∗∗∗p < 0.01. Each panel presents the results from OLS regressions for a different dependent variable, while each column presents the results when changing the fixed effect structures adopted. All dependent variables have been standardized.

### **S11 Table. Effect of the war for sample restricted to online auctions only.**


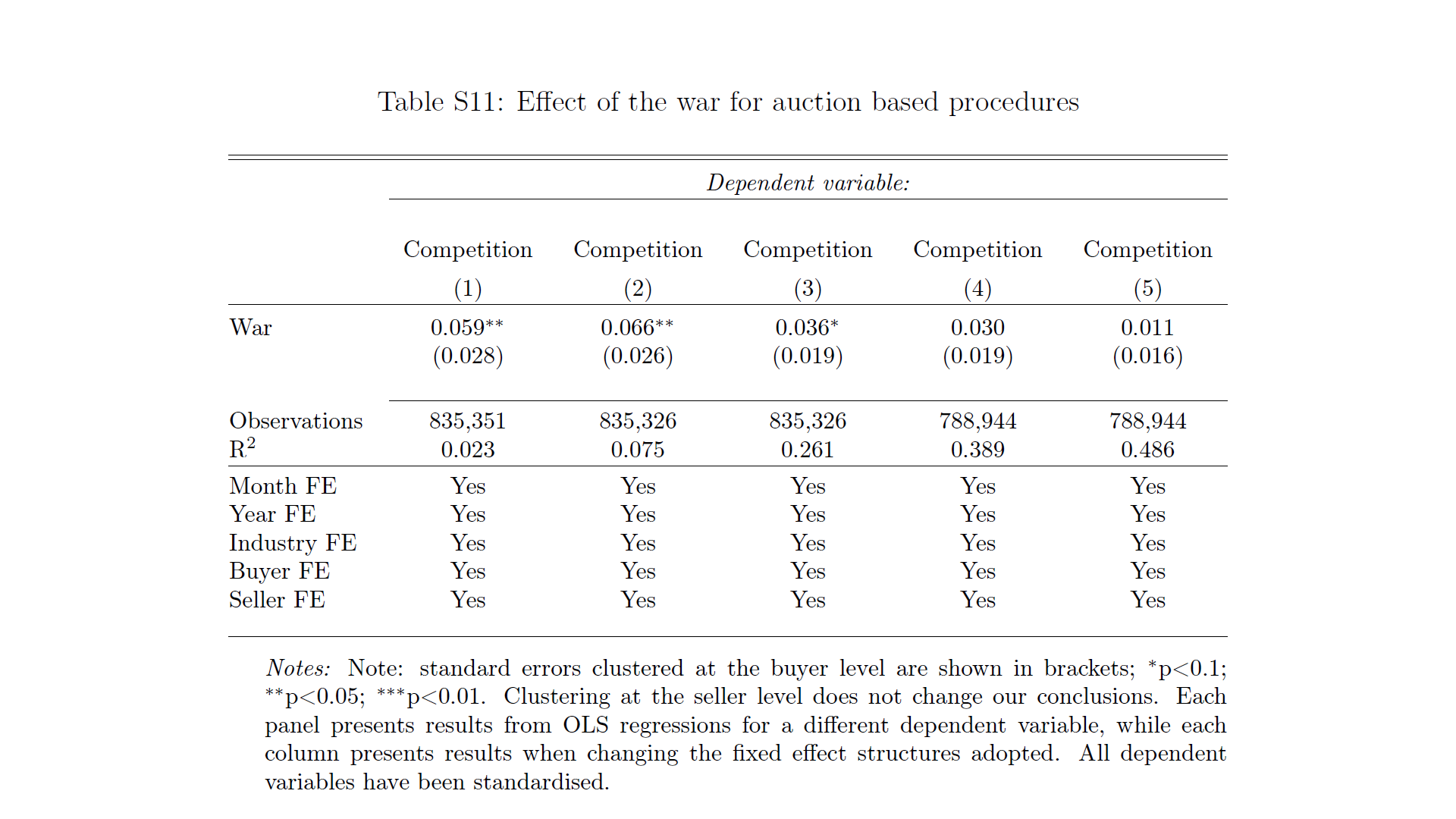


Standard errors clustered at the buyer level are shown in brackets; ∗p < 0.1; ∗∗p < 0.05; ∗∗∗p < 0.01. Each column presents the results from OLS regressions for a different dependent variable while restricting the sample to online auctions only. Full fixed effects are included. All dependent variables have been standardized.

### **S12 Table. Effect of the war for separate samples by types of goods purchased.**


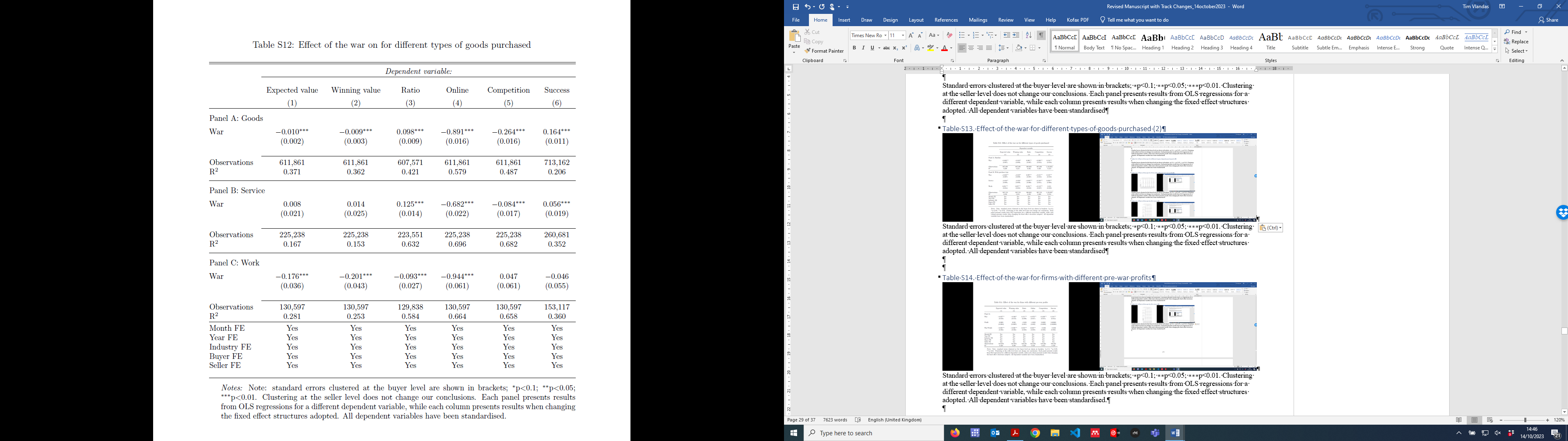


Standard errors clustered at the buyer level are shown in brackets; ∗p < 0.1; ∗∗p < 0.05; ∗∗∗p < 0.01. Each column presents the results from OLS regressions for a different dependent variable, while each panel captures a different sample restriction for goods, services, and work, respectively. All regressions included full fixed effects. All dependent variables have been standardized.

### **S13 Table. Effect of the war while controlling for different types of goods purchased.**


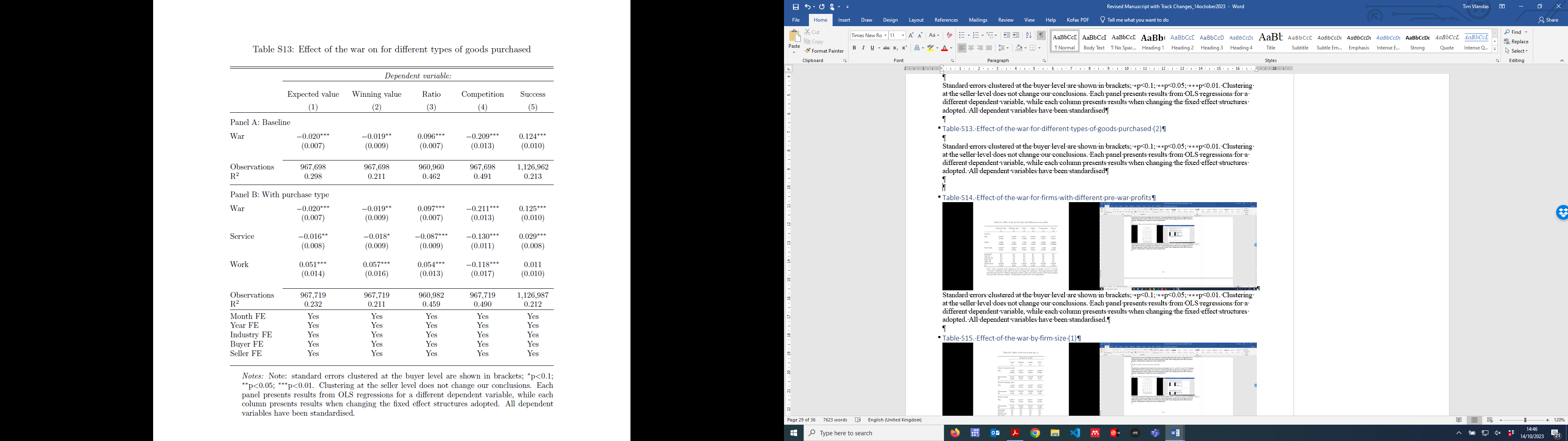


Standard errors clustered at the buyer level are shown in brackets; ∗p < 0.1; ∗∗p < 0.05; ∗∗∗p < 0.01. Each column presents the results from OLS regressions for a different dependent variable. The top panel reproduces our baseline result while the bottom panel controls for type of purchase. Full fixed effects are included. All dependent variables have been standardized.

### **S14 Table. Effect of the war for firms with different pre-war profits.**


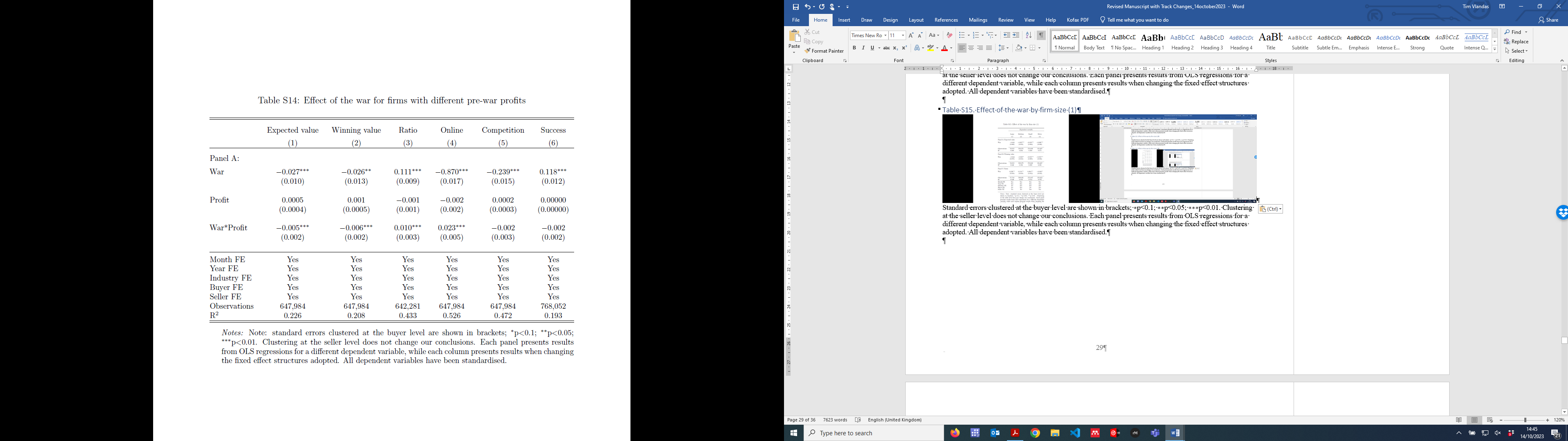


Standard errors clustered at the buyer level are shown in brackets; ∗p < 0.1; ∗∗p < 0.05; ∗∗∗p < 0.01. Each column presents the results from OLS regressions for a different dependent variable. We further include an interaction term between our dummy variable war and a measure of firm profits collected by YouControl. Full fixed effects are included. All dependent variables have been standardized.

### **S15 Table. Effect of the war by firm size (1).**


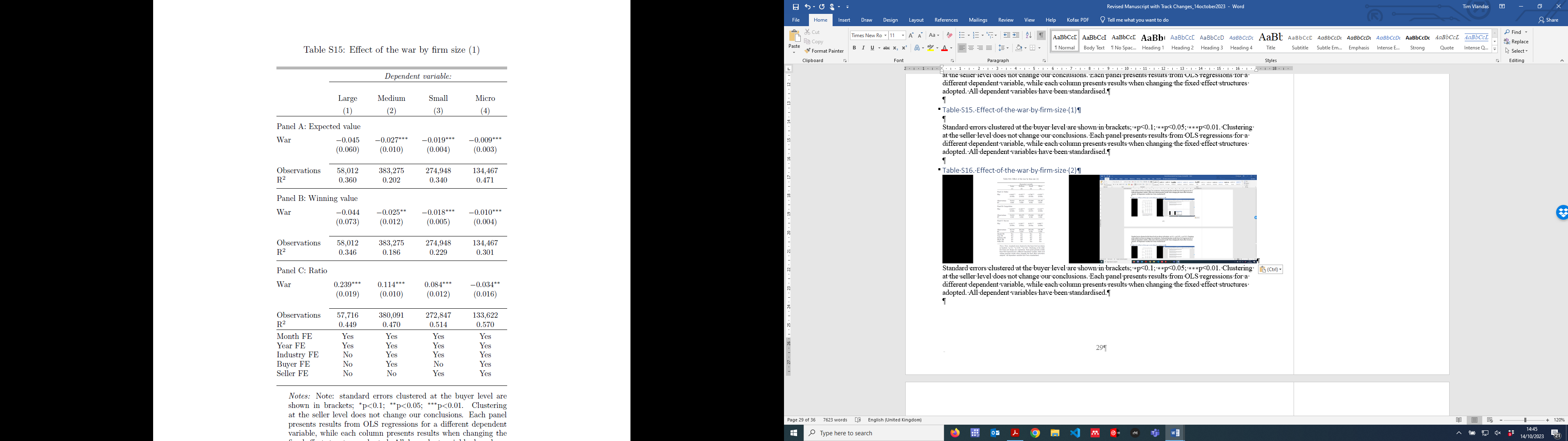


Standard errors clustered at the buyer level are shown in brackets; ∗p < 0.1; ∗∗p < 0.05; ∗∗∗p < 0.01. Each panel presents the results from OLS regressions for a different dependent variable, while each column presents results when changing the fixed effect structures adopted. All dependent variables have been standardized.

### **S16 Table. Effect of the war by firm size (2).**


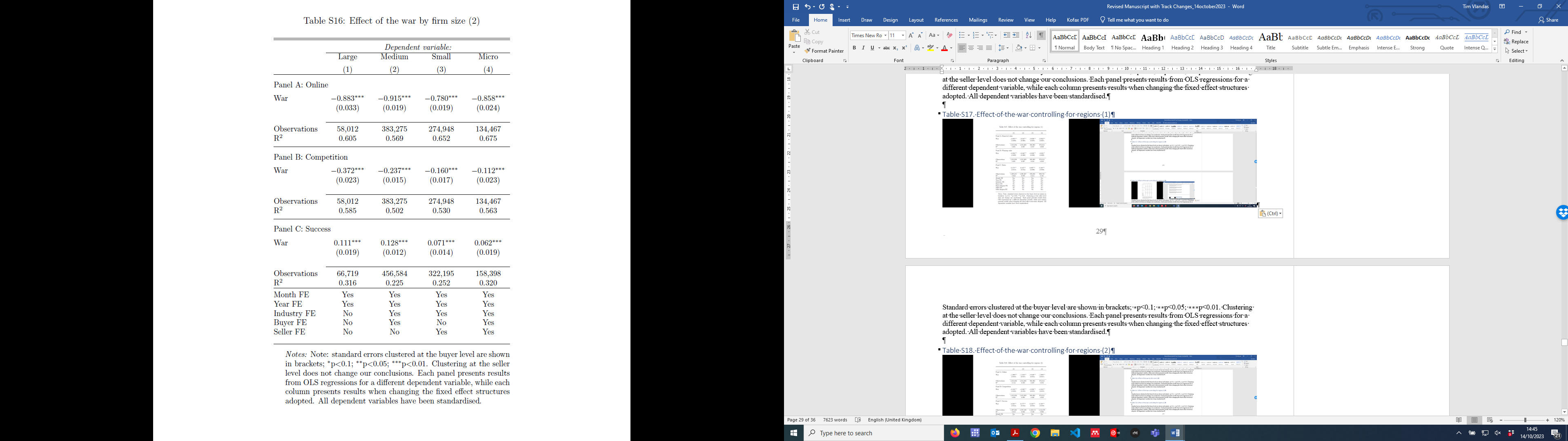


Standard errors clustered at the buyer level are shown in brackets; ∗p < 0.1; ∗∗p < 0.05; ∗∗∗p < 0.01. Each panel presents the results from OLS regressions for a different dependent variable, while each column presents the results when changing the fixed effect structures adopted. All dependent variables have been standardized.

### **S17 Table. Effect of the war controlling for regions (1).**


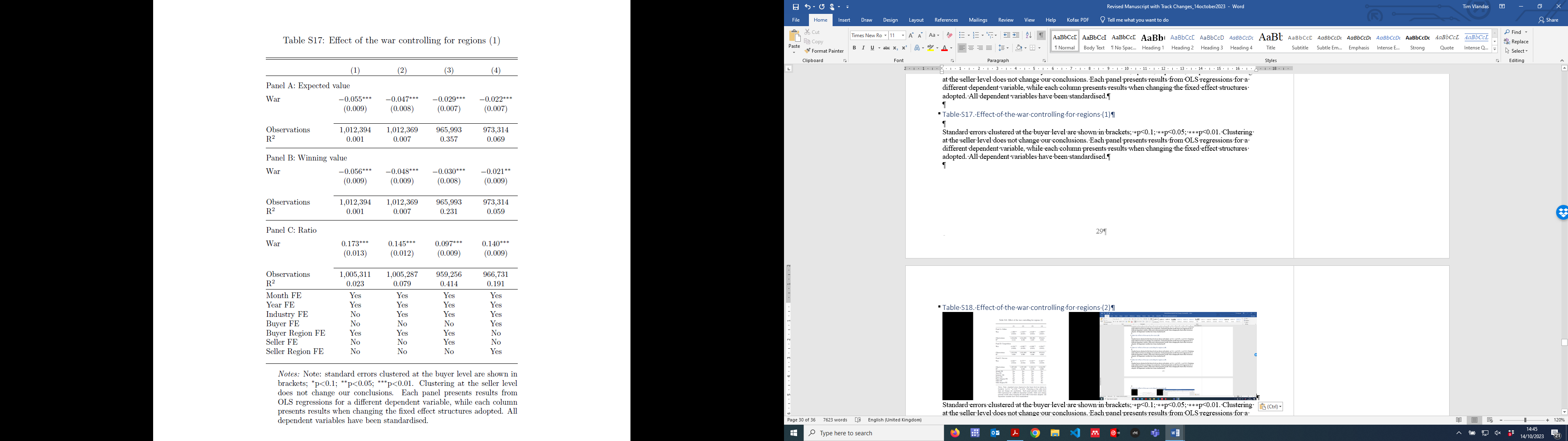


Standard errors clustered at the buyer level are shown in brackets; ∗p < 0.1; ∗∗p < 0.05; ∗∗∗p < 0.01. Each panel presents the results from OLS regressions for a different dependent variable, while each column presents results when changing the fixed effect structures adopted. All dependent variables have been standardized.

### **S18 Table. Effect of the war controlling for regions (2).**


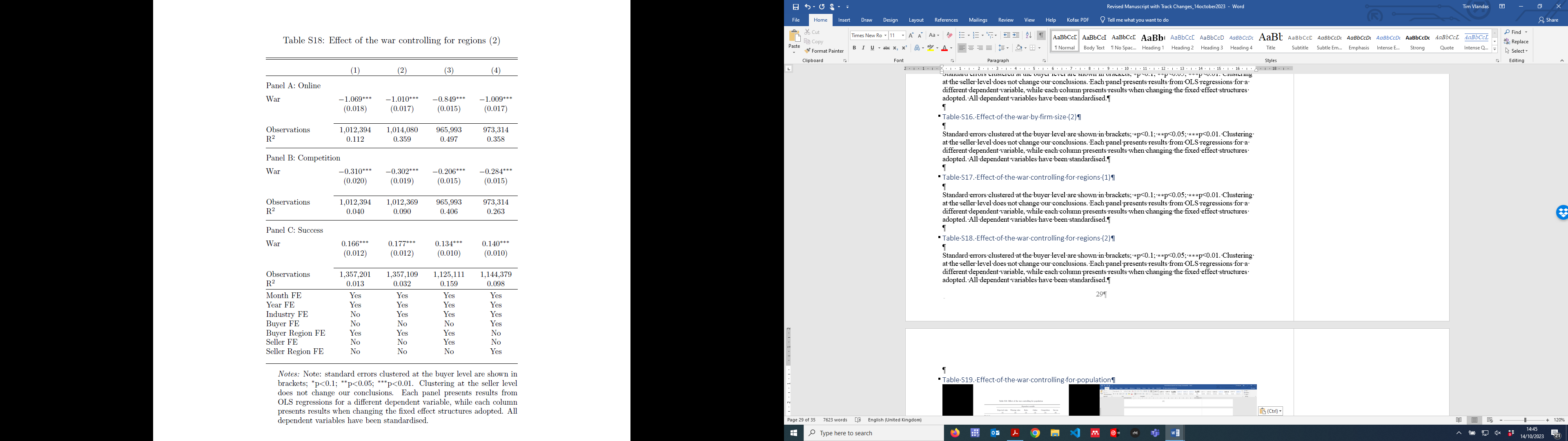


Standard errors clustered at the buyer level are shown in brackets; ∗p < 0.1; ∗∗p < 0.05; ∗∗∗p < 0.01. Each panel presents the results from OLS regressions for a different dependent variable, while each column presents the results when changing the fixed effect structures adopted. All dependent variables have been standardized.

### **S19 Table. Effect of the war controlling for region population.**


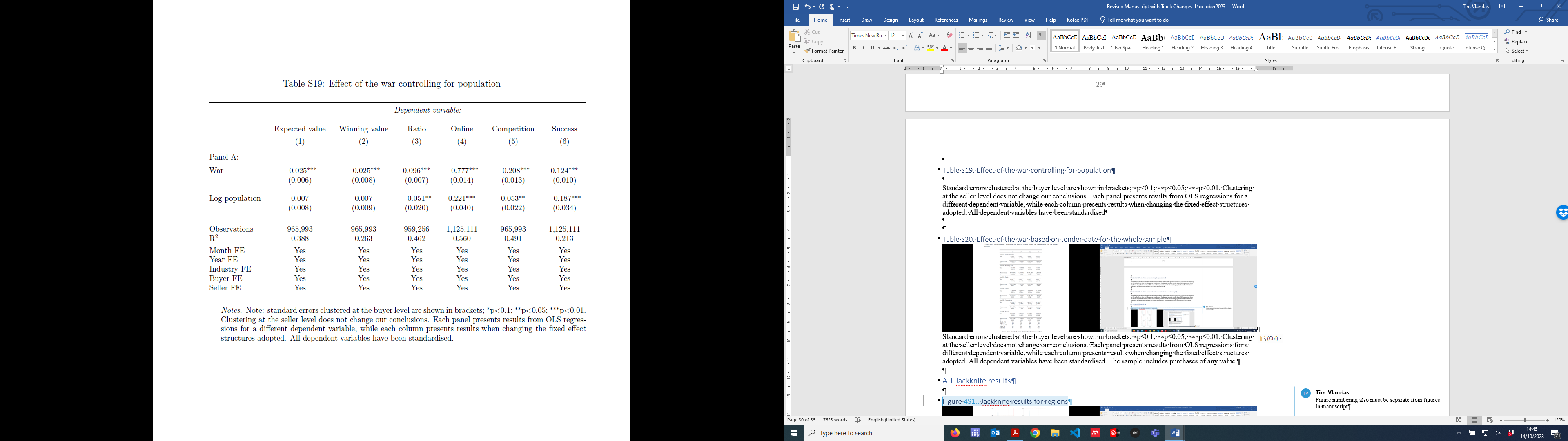


Standard errors clustered at the buyer level are shown in brackets; ∗p < 0.1; ∗∗p < 0.05; ∗∗∗p < 0.01. Each panel presents the results from OLS regressions for a different dependent variable, while each column presents the results when changing the fixed effect structures adopted. All dependent variables have been standardized. This table controls for region population to account for the potential non-linear scaling of procurement activity with population size [1].

References

1 Curado A, Damásio B, Encarnação S, Candia C, Pinheiro FL (2021) Scaling behavior of public procurement activity. PLOS ONE 16(12): e0260806. <https://doi.org/10.1371/journal.pone.0260806>

### **S20. Table. Effect of the war based on the tender date for the whole sample.**


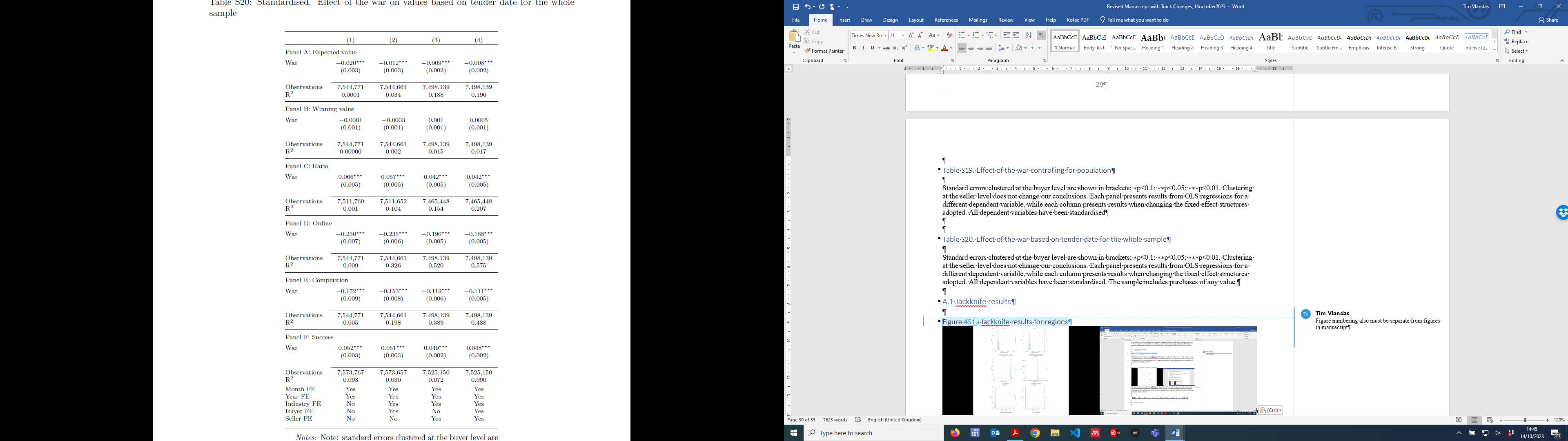


Standard errors clustered at the buyer level are shown in brackets; ∗p < 0.1; ∗∗p < 0.05; ∗∗∗p < 0.01. Each panel presents the results from OLS regressions for a different dependent variable, while each column presents the results when changing the fixed effect structures adopted. All dependent variables have been standardized. The sample includes purchases of any value.

## **S1.1 Jackknife results**


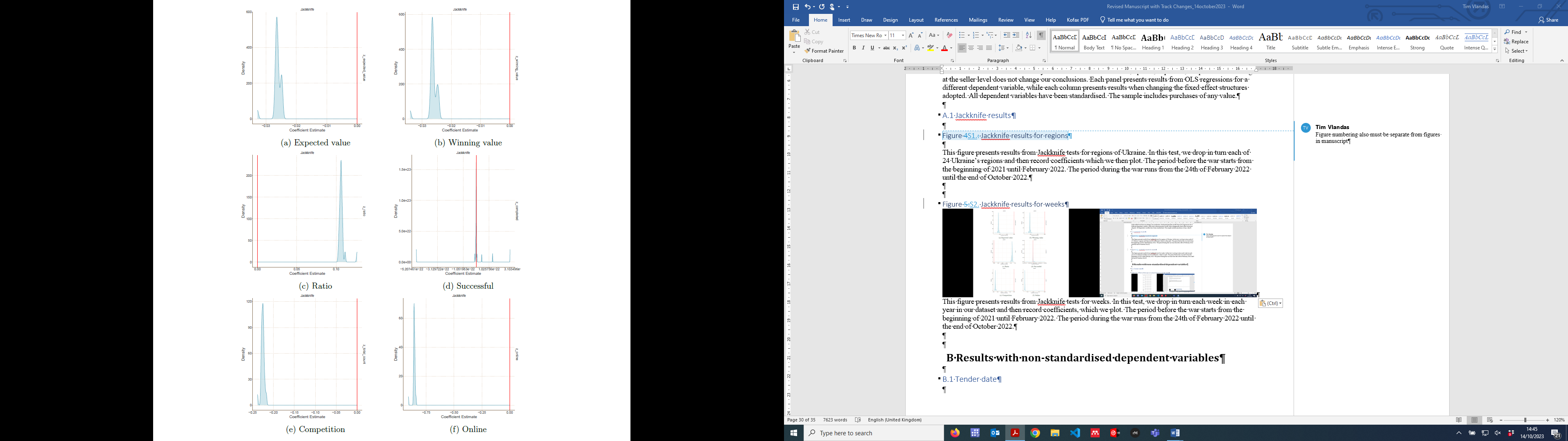


### **S1 Fig. Jackknife results for regions.** Jackknife test results for regions in Ukraine. We drop each of the 24 Ukraine regions in turn to determine the coefficients. The pre-was period is from January 2021 until February 2022, while the post-war period is from 24 February 2022 until the end of October 2022.


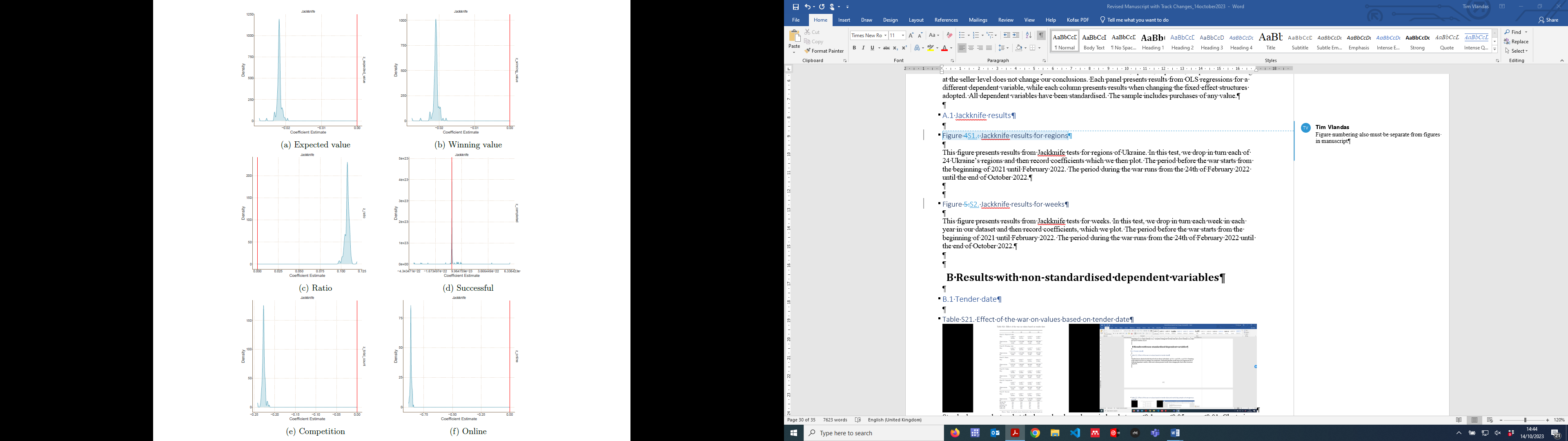


### **S2 Fig. Jackknife results for weeks.** Jackknife test results for weeks. We drop each week in each year in our dataset in turn to determine the coefficients. The pre-was period is from January 2021 until February 2022, while the post-war period is from 24 February 2022 until the end of October 2022.

# S2 Appendix. Results with non-standardized dependent variables

## **S2.1 Tender date**

### **S21 Table. Effect of the war**


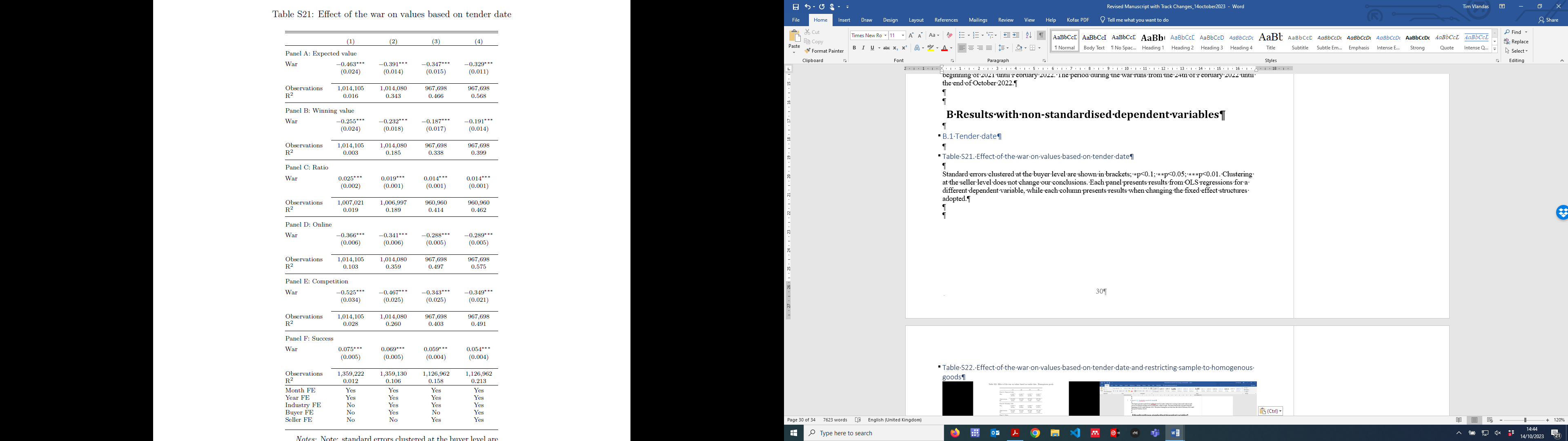


Standard errors clustered at the buyer level are shown in brackets; ∗p < 0.1; ∗∗p < 0.05; ∗∗∗p < 0.01. Each panel presents the results from OLS regressions for a different dependent variable, while each column presents the results when changing the fixed effect structures adopted.

### **S22 Table. Effect of the war, restricting samples to homogenous goods.**


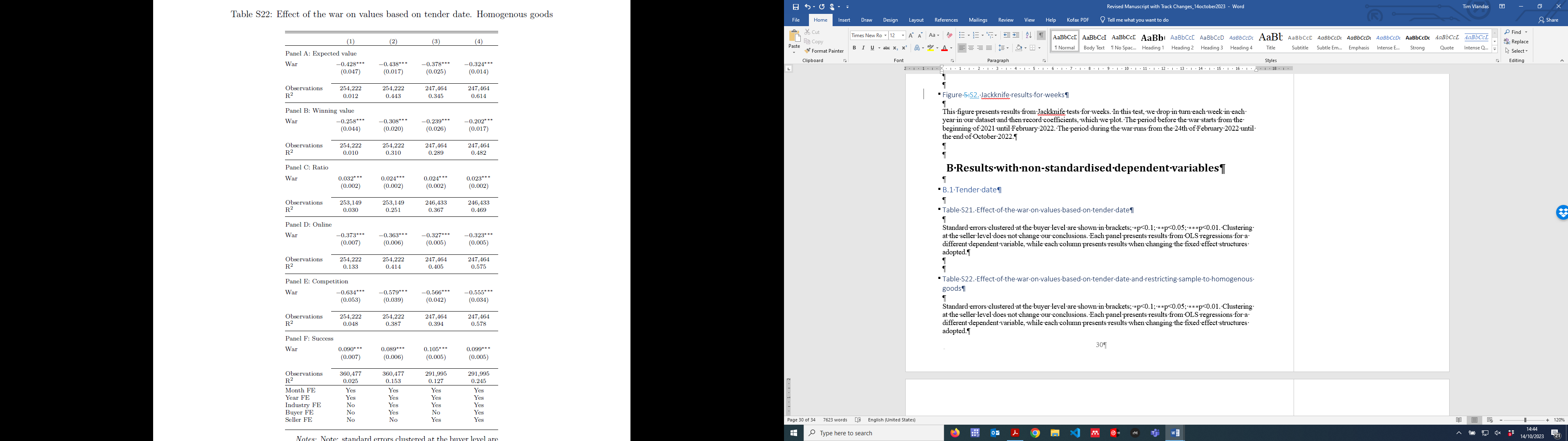


Standard errors clustered at the buyer level are shown in brackets; ∗p < 0.1; ∗∗p < 0.05; ∗∗∗p < 0.01. Each panel presents the results from OLS regressions for a different dependent variable, while each column presents the results when changing the fixed effect structures adopted.

## **S2.2 Publishing date**

### **S23 Table. Effect of the war on values based on publishing date.**


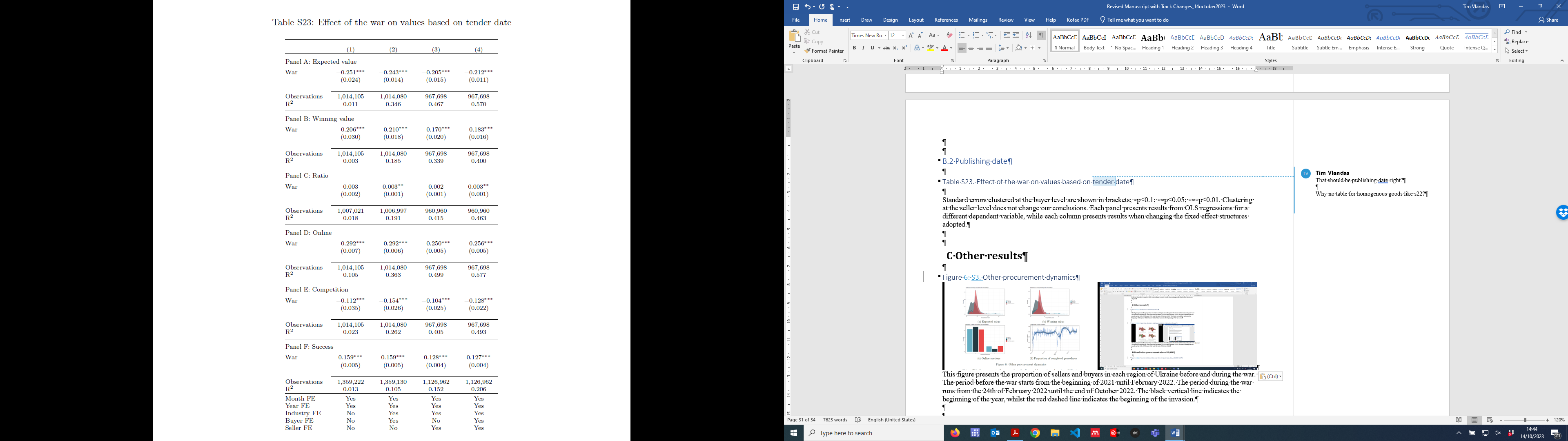


Standard errors clustered at the buyer level are shown in brackets; ∗p < 0.1; ∗∗p < 0.05; ∗∗∗p < 0.01. Each panel presents results from the OLS regressions for a different dependent variable, while each column depicts the results when changing the fixed effect structures adopted. Every procurement transaction has two reference dates: the tender date captured when the purchase occurs; and the publishing date referring to the time the buyer posted the call for tender. Our baseline regressions throughout the analyses use the tender dates as the reference time, and we reproduce our analyses with the publication date in this regression.

# S3 Appendix. Other results


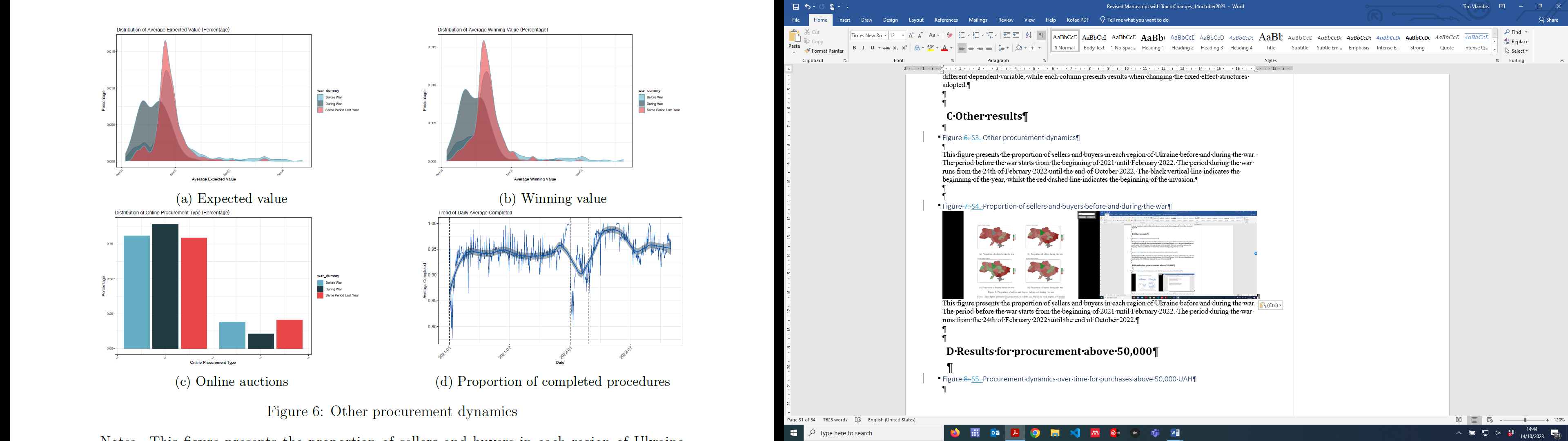


### **S3 Fig. Other procurement dynamics.**


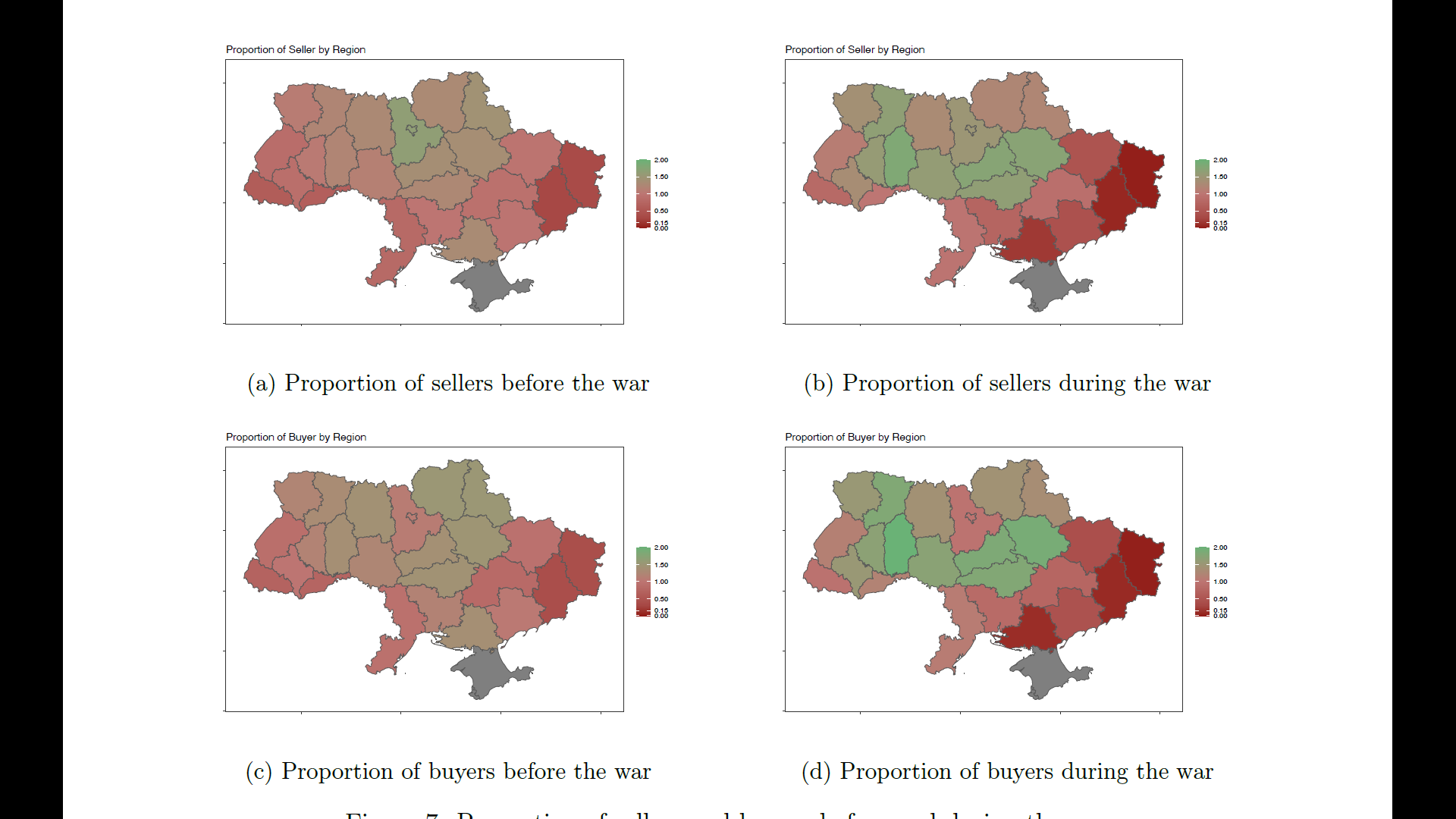


### **S4 Fig. Proportion of sellers and buyers before and during the war (weighed by regional population).** Proportion of sellers and buyers in each region of Ukraine before and during the war, weighed by the population proportion in each of these regions, respectively. The pre-was period is from January 2021 until February 2022, while the post-war period is from 24 February 2022 until the end of October 2022.


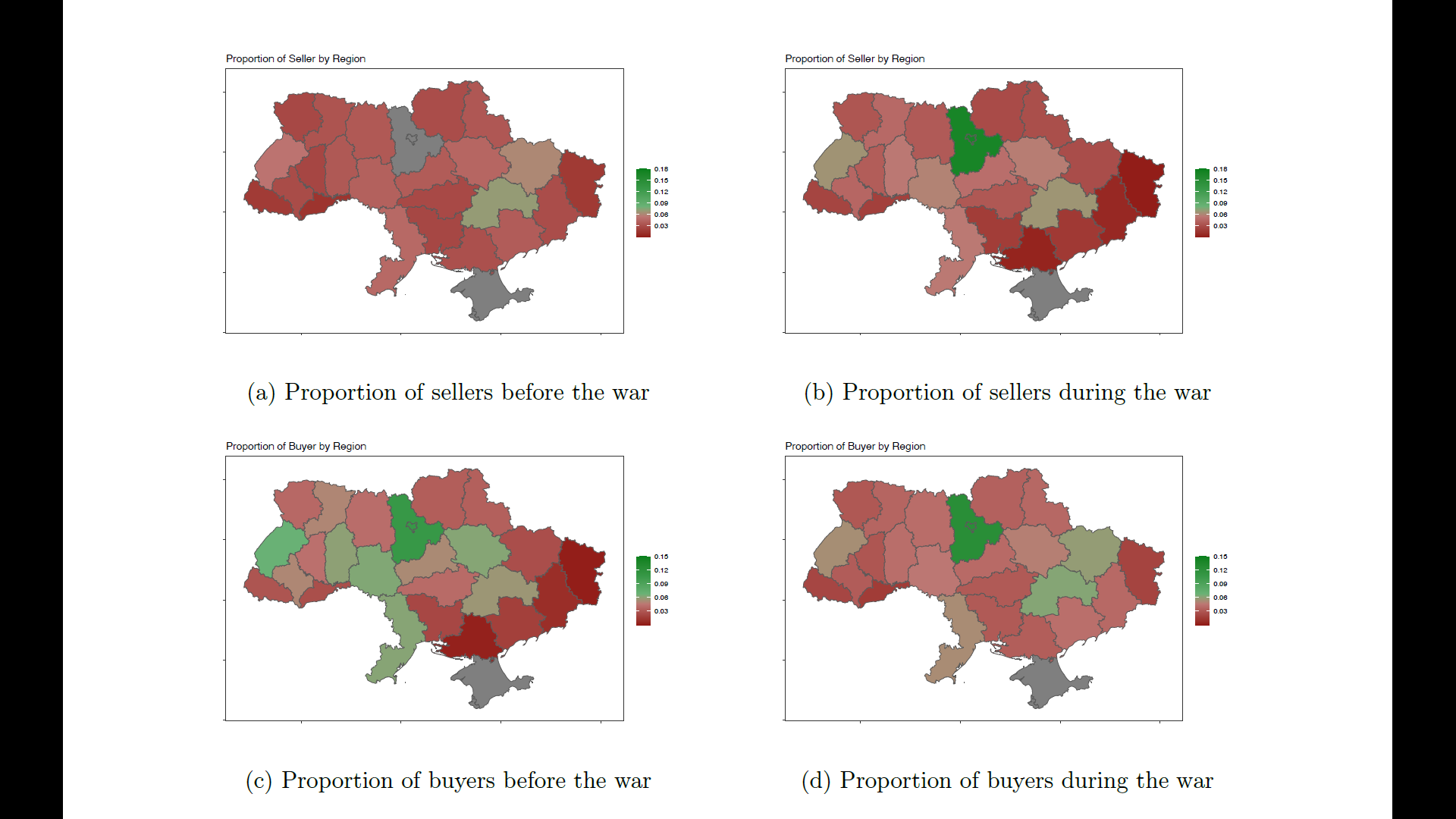


### **Fig S5. Proportion of sellers and buyers before and during the war.** Proportion of sellers and buyers in each region of Ukraine before and during the war. The pre-was period is from January 2021 until February 2022, while the post-war period is from 24 February 2022 until the end of October 2022.

# S4 Appendix. Results for procurement above 50,000


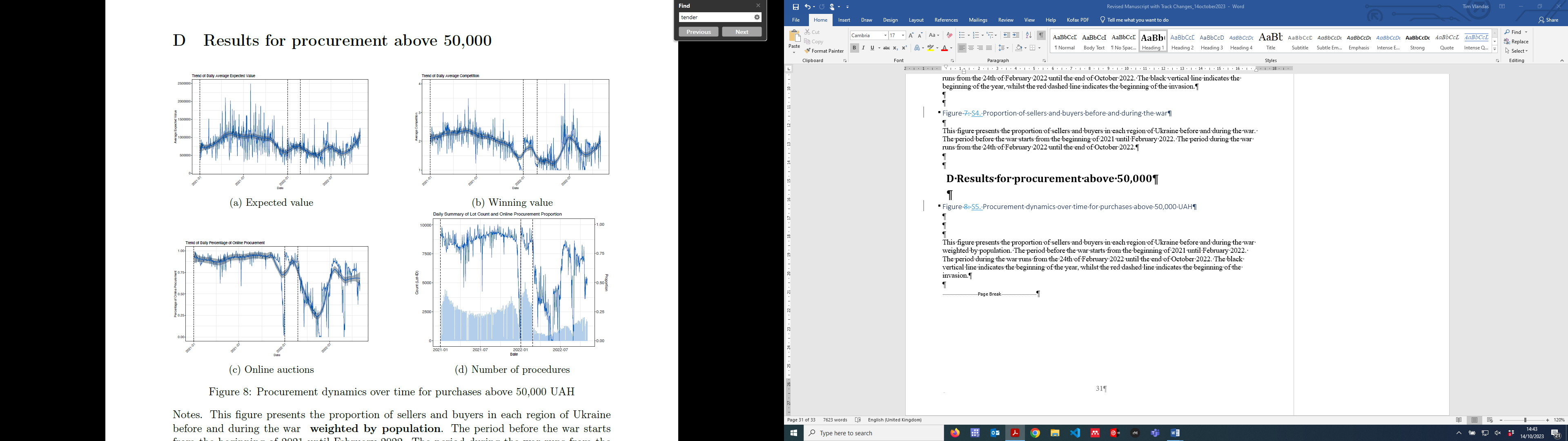


**S6 Fig. Procurement dynamics over time for purchases above 50,000 UAH.** Proportion of sellers and buyers in each region of Ukraine before and during the war weighted by population. The pre-was period is from January 2021 until February 2022, while the post-war period is from 24 February 2022 until the end of October 2022. The black and vertical lines indicate the beginning of the year and the invasion, respectively.

# S5 Appendix. Procurement system

Ukraine has initiated significant efforts to reform its procurement legislation since 1997 in an attempt to attain the standards of the World Trade Organization and the European Union. While partly successful, these reform attempts have created excessively complicated rules with too many loopholes, particularly considering the corruption problems that Ukraine faced at the time. This complex regulatory framework in turn hindered investments and private sector efficiency. Between November 2013 and 2014, the Ukrainian government's decision to halt plans for closer integration with the European Union led to the so-called Euromaidan protests, which ultimately brought about the removal from power of President Yanukovich.

These developments unleashed an unprecedented wave of political and market reforms aimed at improving the procurement sector. A group of Euromaidan volunteers, the Ministry of Economic Development and Trade, and private sector stakeholders collaborated on a novel procurement platform in 2014 to combat corruption and increase transparency. This new procurement platform, introduced in February 2015, was denoted as "Prozorro", derived from the Ukrainian word for "transparent". The main aim of the platform was to improve the efficiency and transparency of public procurement procedures and strengthen the connections between the government, private sector, and civil society. After the adoption of a new public procurement law in December 2015, Prozorro became mandatory for all public agencies in August 2016. The result was an exceptional level of transparency with all government procurement transactions fully accessible on the platform for the wider public to access in real time. Indeed, the platform received significant recognition, including major international government procurement awards such as the World Procurement Award and the Open Government Awards in 2016 and 2021. The 2015 procurement law specified that certain goods and services would not be required to be reported on the platform. These include: the production of banknotes, coins, state honors, and official documents such as passports and national identification; matters pertaining to national security and defense; activities associated with the servicing and repayment of public debt; management of gold and foreign exchange reserves; procurement or leasing of land, buildings, other real estate, or property; services provided by international arbitration courts and international commercial arbitrations for disputes involving government agencies; services rendered by financial organizations, including the provision of loans, guarantees, financial leasing, and other financial services; financial services related to the issuance, purchase, sale, or transfer of securities or other financial instruments; services delivered by the National Bank of Ukraine; acquisitions made by foreign diplomatic organizations; goods and services requested by government ministries responsible for the formulation and execution of state healthcare policies; goods and services whose prices are sanctioned by central government authorities.

Depending on the expected purchasing value, a government authority can generally make two types of purchases: auctions which must be executed directly on the platform and are available in real time; and reports that are used for small-value purchases, where the supplier is chosen by the procurement office and must then release all information on the procurement platform. In addition, government authorities can undertake negotiation procurement whereby they first meet potential sellers to narrow down the specifications of the purchase and the purchase subsequently takes place as auction on the platform. This procurement type is not common and only available for certain purchases (e.g. software). Therefore, in the period before the invasion started, all purchases regardless of whether they were reports or auctions, must have been run through the platform. In contrast, following the invasion, the government of Ukraine listed different mandatory reporting requirements: procurement officers were allowed to report purchases at their discretion during the war period but were informed that they would be required to report all purchases after the war ended. In June 2022, the government changed the requirements again: for purchases with an expected value under 50,000 UAH, the purchase could continue to take place without using an electronic procurement system, whereas purchases worth more than 50,000 UAH must be made through the Prozorro system. To ensure that our analyses are not biased by these two changes, we conduct two robustness checks. First, while our baseline analyses were restricted to purchases above 50,000 UAH to maximize reporting requirements during the war, we also reran our analyses without this threshold restriction and the results were unchanged. Second, we ensured that our results were robust to the inclusion of a dummy variable taking that equals one when the aforementioned policy change was introduced and 0 otherwise. As we discuss in the robustness checks in our manuscript this also did not change our results.
